# Arsenate immobilisation and mimetite formation on Pb-modified zeolite under variable aqueous chemistry

**DOI:** 10.1007/s10653-026-03472-6

**Published:** 2026-09-14

**Authors:** Ewa Stępień, Frederik Dunkel, Maciej Manecki, Tomasz Bajda

**Affiliations:** 1 Faculty of Geology, Geophysics and Environmental Protection, AGH University of Krakow, al. Mickiewicza 30, 30-059 Kraków, Poland; 2 Chair of Geology and Economic Geology, Department Applied Geosciences and Geophysics, Montanuniversität Leoben, Peter-Tunner-Straße 5, 8700 Leoben, Austria

**Keywords:** Arsenate immobilisation, Mimetite, Pb-modified clinoptilolite, Aqueous chemistry, Mineral precipitation, Contaminant sequestration

## Abstract

Arsenic contamination in water remains a global challenge, and understanding the mineralogical controls on arsenate sequestration is essential for predicting its behaviour in natural and engineered aqueous systems. This study investigates arsenate immobilisation on Pb-modified clinoptilolite through the precipitation of mimetite (Pb_5_(AsO_4_)_3_Cl), a sparingly soluble apatite-group mineral. The Pb-modified zeolite (Na-exchanged clinoptilolite containing ~ 70 g Pb/kg), was reacted with As(V) solutions (0.1–50 mg/L) containing 20 mg/L Cl^−^ across pH 2–8 for up to 168 h. Arsenate concentrations decreased rapidly, with measurable uptake within 1 min and up to 99% immobilisation achieved within 1 h at pH 6–8. Lower immobilisation at pH 2–4 reflects reduced mimetite stability and pH-dependent arsenate speciation. Mimetite precipitated directly at the solid-solution interface through the reaction of surface-released Pb^2+^ with arsenate and chloride, indicating an interface-controlled process driven by local supersaturation. The mechanism was largely unaffected by competing cations (K^+^, Mg^2+^, Ca^2+^, Al^3+^, Mn^3+^, Cu^2+^, Ni^2+^, Zn^2+^, Cd^2+^) and anions (F^−^, SO_4_^2−^, PO_4_^3−^). Arsenate immobilisation in natural waters from Bad Wildbad (Germany), Złoty Stok (Poland), and the Upper Mur Valley (Austria) varied substantially with solution composition, ranging from 51 to 99%. These results demonstrate that arsenate sequestration in this system is governed by coupled surface-mediated Pb release and secondary mineral precipitation, providing new insight into the controls on arsenic mobility in Pb-bearing environments.

**Supplementary Information:**

The online version contains supplementary material available at https://doi.org/10.1007/s10653-026-03472-6.

## Introduction

Arsenic is a widespread semi-metal occurring in the atmosphere, soils, rocks, natural waters, and living organisms. More than 300 arsenic-bearing minerals have been identified in nature, reflecting its broad geochemical distribution and environmental relevance (Battistel et al., 2021; Drahota & Filippi, 2009; Long et al., 2019a; Podgorski & Berg, 2020; Smedley & Kinniburgh, 2002). Arsenic contamination of water is a global concern because its mobility, speciation, and persistence are strongly controlled by aqueous geochemical conditions. In natural waters, arsenic occurs predominantly in inorganic form as oxyanions of trivalent arsenite [As(III)] or pentavalent arsenate [As(V)]. Their distribution is governed primarily by redox conditions, with As(III) prevailing under reducing environments and As(V) under oxidizing conditions. These species also differ markedly in aqueous behaviour. Arsenite is typically present as undissociated H_3_AsO_3_ up to about pH 9 and is generally more mobile and toxic, whereas arsenate dissociates progressively from H_3_AsO_4_ to H_2_AsO_4_^−^, HAsO_4_^2−^, and AsO_4_^3−^ with increasing pH and is generally more amenable to immobilisation by adsorption or precipitation. Organic arsenic species are generally less toxic and are not considered in the present study (Chaudhary et al., 2024; Long et al., 2019a; Mandal & Suzuki, 2002; Mohan & Pittman, 2007; Mojiri et al., 2024; Nazari et al., 2017; Smedley & Kinniburgh, 2002).

Arsenic enters aquatic systems through both natural and anthropogenic pathways. Natural sources include mineral weathering, volcanic emissions, and biological activity, whereas anthropogenic inputs arise from mining, fossil fuel combustion, and historical uses in wood preservation, pesticides, and herbicides (Mohan & Pittman, 2007; Podgorski & Berg, 2020; Smedley & Kinniburgh, 2002). Elevated inorganic arsenic concentrations in water pose significant risks to ecosystems and human health, with exposure linked to skin lesions, vascular and neurological disorders, and several forms of cancer (Alka et al., 2021; Chaudhary et al., 2024; Mojiri et al., 2024; Podgorski & Berg, 2020). Although drinking water is the principal exposure route for humans, arsenic contamination also affects mine waters, industrial effluents, and other geochemically complex aqueous environments (Alka et al., 2021; Smedley & Kinniburgh, 2002).

The mobility and fate of arsenic in aquatic systems is controlled by a combination of speciation, pH, redox conditions, and interactions with mineral surfaces and dissolved ions. In natural and engineered systems, arsenic is commonly immobilized through adsorption, ion exchange, or precipitation of sparingly soluble phases (Chaudhary et al., 2024; Magalhães, 2002; Mohan & Pittman, 2007; Mojiri et al., 2024). Consequently, a variety of remediation strategies have been developed, including adsorption onto iron- and aluminium-based materials (e.g., granular ferric hydroxide and iron oxyhydroxides; Banerjee et al., 2008; Driehaus et al., 1998; Guan et al., 2008; Szlachta & Wójtowicz, 2016) or zero-valent iron-based treatments (Bang et al., 2005; Kanel et al., 2005; Lackovic et al., 2000; Wang et al., 2017), membrane-based separation processes (Nicomel et al., 2016; Shih, 2005), electrochemical methods (Ali et al., 2011; Syam Babu & Nidheesh, 2021), phytoremediation (Roy et al., 2015; Vithanage et al., 2012), and stabilization/solidification techniques (e.g. vitrification; Wang et al., 2025).

Among these pathways, precipitation is particularly important because it partitions dissolved arsenic into solid phases with low aqueous solubility, and is used extensively due to its simplicity and low cost. A variety of arsenic-bearing minerals have been investigated for this purpose, including Fe(III) arsenates such as scorodite (FeAsO_4_·2H_2_O) (Krause and Ettel, 1989; Lu et al., 2019; Twidwell et al., 2005; Wang et al., 2019) or calcium arsenates, e.g. johnbaumite (Ca_5_(AsO_4_)_3_OH; Bothe & Brown, 1999; Moon et al., 2004; Martínez-Villegas et al., 2013; Zhang et al., 2019).

However, the long-term stability of such phases depends strongly on their composition, structure, and surrounding geochemical conditions. These considerations highlight the importance of identifying arsenic-bearing minerals with well-defined crystal chemistry and predictable geochemical behaviour (Alka et al., 2021; Long et al., 2019a; Nazari et al., 2017). One such phase is mimetite, Pb_5_(AsO_4_)_3_Cl, a sparingly soluble apatite-group mineral whose low solubility and structurally constrained composition make it a potentially important sink for arsenate in chloride-bearing systems (Bajda, 2010; Magalhães & Silva, 2003; Markl et al., 2014; Solecka et al., 2025).

Substrate-mediated mineral precipitation may strongly influence how such phases nucleate and develop. In porous aluminosilicate systems, cation-exchanged surfaces can provide both a localized source of reactive metal ions and an interface favourable for heterogeneous nucleation. Natural clinoptilolite ((Na/Ca/K)_3−6_[Al_6−7_Si_29−30_O_72_]·20H_2_O) is particularly suitable for investigating this behaviour because of its high cation-exchange capacity, structural stability, and widespread occurrence. In Pb-bearing form, it provides a useful model system for examining the coupling between Pb release, arsenate speciation, and mimetite formation in the presence of chloride.

Although lead chloroarsenate (mimetite) precipitation has previously been proposed as a possible route for arsenic sequestration (e.g. Bajda, 2010; Bajda et al., 2007; Comba, 1987; Flis et al., 2011; Kleszczewska-Zębala et al., 2016; Long et al., 2019b; Magalhães, 2002; Magalhães & Silva, 2003), the aqueous geochemical controls governing this process remain insufficiently constrained. Recent work by our group demonstrated the feasibility of using Pb-modified zeolite as a reactive material for arsenate immobilisation through coupled Pb release and mimetite precipitation (Stępień et al., 2025). In this approach, the zeolite acts as a Pb reservoir, supplying Pb^2+^ to the aqueous phase through desorption processes, while the released Pb reacts with arsenate and chloride ions to promote the precipitation of mimetite (Pb_5_(AsO_4_)_3_Cl), according to:

$$5{\mathrm{Pb}}^{2 + } + \, 3{\mathrm{AsO}}_{4}^{3 - } \, + {\text{ Cl}}^{ - } \to {\text{ Pb}}_{5} \left( {{\mathrm{AsO}}_{4} } \right)_{3} {\mathrm{Cl}}$$

This initial study established the proof of concept for mimetite formation in the presence of Pb-modified clinoptilolite under selected conditions; however, the factors controlling the efficiency, stability, and environmental applicability of this process remain unclear. In particular, the effects of pH, arsenate concentration, reaction kinetics, and aqueous composition on Pb availability, mimetite formation, and arsenate immobilisation have not been systematically evaluated. Therefore, the present study extends this initial concept by investigating substrate-mediated mimetite precipitation under a broader range of environmentally relevant conditions, including synthetic solutions and natural arsenic-bearing waters.

The objectives of this study were to determine the geochemical conditions under which mimetite formed, to characterize the resulting solids, and to evaluate how aqueous composition influenced arsenate partitioning, Pb behaviour, and solid-phase development in both synthetic and natural waters. By linking aqueous geochemistry with solid-phase evolution, this study aimed to clarify the mechanisms and environmental controls governing substrate-mediated arsenate mineralization.

## Materials and methods

### Chemicals and solutions

Batch experiments were carried out under ambient laboratory conditions (~ 25 °C) in open air, using polyethylene test tubes without temperature regulation. Double-distilled water and analytical-grade reagents supplied by Thermo Scientific Inc., Chempur Inc., and POCH Inc. were employed.

Sodium arsenate dibasic heptahydrate (Na_2_HAsO_4_·7H_2_O) served as the source of arsenate ions, while sodium chloride (NaCl) was used to introduce chloride ions. Lead ions were provided as lead nitrate Pb(NO_3_)_2_. Additional metal cations (Mg, Al, K, Ca, Mn, Ni, Cu, Zn, and Cd) were added in the form of their nitrate salts. Anions, including sulphate (SO_4_^2−^), phosphate (PO_4_^3−^), and fluoride (F^−^), were introduced as their corresponding sodium salts. The pH of the solutions was adjusted by the gradual addition of 0.5 or 1 M HNO_3_ and 0.1 or 0.5 M NaOH using a single-use pipette. Measurements of pH were performed using a pH-meter ELMETRON CPC-401, calibrated with standard buffer solutions.

### Natural waters sampling

Sampling of natural waters was carried out at three sites between May 2024 and August 2025. In May 2024, water was collected in Germany from the technical facility at Vital Therme (Bad Wildbad; Fig. SM 2). In November 2024 and April 2025, samples were taken from the Trująca River in Złoty Stok, Poland (Fig. SM 3), and in August 2025 from two locations in a historic copper–gold mining district within the Upper Mur Valley (in the vicinity of Knittelfeld, Austria; Fig. SM 4). For each site, a few litres of water were collected. On-site, portions of the samples were filtered through 0.2 μm pore-sized syringe filters and stored in 50 mL polypropylene tubes acidified with 2% HNO_3_ for chemical analysis. In parallel, unfiltered water samples were collected in polypropylene bottles for additional analyses and laboratory experiments. Throughout sampling, transport, storage, and preparation, precautionary measures were taken to minimize the risk of contamination. Samples were transported and stored under refrigerated conditions (approximately 4 °C) until further processing and analysis.

The sampling sites represent contrasting geological and hydrogeochemical settings. The thermal waters of Bad Wildbad (Northern Black Forest; coordinates: 48° 44′ 57″ N, 8° 32′ 55″ E) are meteoric-derived fluids that circulated to depth through fractures in the crystalline basement and ascended along fault zones associated with the Nordschwarzwald. Interaction with metamorphic and crystalline rocks (including localized sulphide-bearing horizons) and deep fluid-rock interaction are plausible natural sources of trace elements, including arsenic, through oxidation of sulphides and leaching from accessory minerals (Banning, 2021; Plinninger & Thuro, 1998; Stober et al., 2025).

The Trująca River drains a historically mined catchment (Złoty Stok; coordinates: 50° 26′ 21″ N, 16° 52′ 28″ E), long known for gold and arsenic production; mine wastes, tailings and exposed arsenic-bearing ores are documented sources of high arsenic concentration in river waters and sediments. Surface water arsenic in this basin has been extensively reported as elevated (often exceeding drinking-water limits) due to weathering and transport of mine residues and contaminated Quaternary sediments. This site therefore represents a mining-impacted, oxidizing surface-water environment useful for testing sequestration under realistic contaminated conditions (Karczewska et al., 2007; Marszałek & Wąsik, 2000; Siuda & Macioch, 2018).

At the third sampling site in the Upper Mur Valley, mining for copper and gold has been carried out predominantly in the eighteenth and nineteenth century, leaving behind numerous mine adits and small waste rock piles. As-bearing minerals such as Arsenopyrite (FeAsS) are present in the historically mined orogenic-type sulphide mineralization, with the waste rock piles also containing up to 0.6% As (Dunkel et al., 2025). The Upper Mur Valley samples were taken at two locations directly affected by the former mining activities, with one sample representing oxidized surface water downstream of As-bearing waste rock piles (Upper Mur Valley A; coordinates: 47° 14′ 10″ N, 14° 44′ 25″ E) and the other oxygen-poor water originating from a flooded mine gallery (Upper Mur Valley B; coordinates: 47° 14′ 25″ N, 14° 44′ 10″ E).

### Examination methods and equipment

As(V) concentration was determined using the molybdenum blue colorimetric method (Lenoble et al., 2003) with a HITACHI U-800 UV–Vis spectrophotometer. The colour reagent consisted of ammonium molybdate ((NH_4_)_6_Mo_7_O_24_·4H_2_O), potassium antimonyl tartrate (K(SbO)C_4_H_4_O_6_·0.5H_2_O), and concentrated H_2_SO_4_, with ascorbic acid (10% solution) used as the reducing agent. Measurements were performed in absorbance mode at 870 nm using 10 mm polystyrene cuvettes. Colour development was carried out at ambient temperature (~ 22 °C) for 45 min. Calibration standards ranged from 0.01 to 5 mg As(V)/L, yielding an R^2^ > 0.999. Analytical blanks were included in each batch. The method detection limit (LoD) was 0.02 mg/L, while reliable quantification (LoQ) was achieved for concentrations ≥ 0.05 mg/L.

Sulphate (SO_4_^2−^) concentration was measured using a HACH DR6000 UV–Vis spectrophotometer with the turbidimetric method (SulfaVer 4 reagent pillows). Analyses were performed at 450 nm in 10 mL glass cuvettes, with measurements taken 5 min after reagent addition. The method’s limit of detection (LoD) was 0.4 mg SO_4_^2−^/L, and concentrations ≥ 1.0 mg SO_4_^2−^/L could be reliably measured.

Aluminum concentration was determined using Chrome Azurol S Hach LCK cuvette tests with a HACH DR6000 UV–Vis spectrophotometer. The 13 mm cuvettes contained pre-dosed reagents, including Chromazurol S and stabilizing agents, which form a coloured complex with aluminum ions. Measurements were taken 25 min after reagent mixing at 430 nm. The method detection limit (LoD) was 0.005 mg Al/L, and the limit of quantification (LoQ) was 0.02 mg Al/L.

AAS with an air-acetylene flame was used to determine Mg, K, Ca, Mn, Ni, Cu, Zn, Cd, and Pb concentrations in solution. Measurements were performed with a SavantAA spectrometer (GBC Scientific Equipment) using hollow cathode lamps. The instrument was calibrated with matrix-matched standard solutions: Mg, K, Ca, and Mn standards were prepared from dissolved chemical reagents (Mg(NO_3_)_2_·6H_2_O, KNO_3_, Ca(NO_3_)_2_·4H_2_O, Mn(NO_3_)_2_·4H_2_O), while Pb, Cu, Cd, Zn, and Ni standards were prepared from stock solutions (Sigma Aldrich Inc.) of Pb(NO_3_)_2_, Cu(NO_3_)_2_, Cd(NO_3_)_2_, Zn(NO_3_)_2_, and Ni(NO_3_)_2_. Calibration curves indicated R^2^ > 0.98. Flame conditions were optimized with an acetylene flow of 2.0 L/min, and an air flow of 13.5 L/min. Samples were introduced at a rate of 5 mL/min, with each measurement performed in triplicate. Prior to analysis, all samples were filtered through 0.20 μm cellulose membranes. The method detection limits (LoD) and corresponding analytical wavelengths, arranged by increasing atomic weight, were as follows: Mg - 0.10 mg/L (202.6 nm), Ca - 0.08 mg/L (239.9 nm), K** - **0.08 mg/L (404.4 nm), Mn - 0.07 mg/L (403.1 nm), Ni - 0.09 mg/L (351.5 nm), Cu - 0.03 mg/L (222.6 nm), Zn - 0.40 mg/L (307.6 nm), Cd - 0.02 mg/L (326.1 nm), and Pb - 0.01 mg/L (217.0 nm). Data were processed using the GBC software and manually verified using Microsoft Excel.

Concentrations of arsenic (As) and lead (Pb) in some of the investigated samples (subsection 2.4.1 Influence of pH and initial As concentration) were determined using an ICP-OES spectrometer (Perkin Elmer Optima 7300DV). In samples where arsenic and phosphorus were present simultaneously (subsection 2.4.5 Effect of accompanying cations and anions; effect of PO_4_^3−^ addition), phosphorus (P) concentrations were also measured using the same method. All analyses were performed at the Laboratory of Hydrogeochemistry, Department of Hydrogeology and Engineering Geology, AGH University of Krakow—an accredited testing facility. The laboratory’s scope of accreditation includes chemical testing and sampling of water, covering As (0.005–1 mg/L), Pb (0.005–10 mg/L), and P (0.01–5 mg/L) in compliance with PN-EN ISO 11885–2009 and relevant PN-ISO sampling standards. 

The mineral composition of the experimental material and the reaction products was determined by XRPD using a Rigaku SmartLab diffractometer (Rigaku Corporation, Japan), equipped with a rotating Cu anode X-ray tube (Cu Kα radiation, λ = 1.5406 Å) and a theta-theta goniometer with a 30 cm radius and 0.5–150° 2θ measurement range. Powder samples, previously ground in an agate mortar, were mounted on zero-background silicon wafer holders. Diffraction data were collected using a scintillation and strip detector (SC and D/tex Ultra) over a 2θ range of 2°–75° with a step size of 0.05° and a counting time of 1 s per step. Phase identification was performed by matching interplanar spacings and peak intensities with standard reference patterns from ICDD PDF database, processed using XRAYAN software (ICDD, PDF-2 Database, 2025; Marciniak et al., 2006).

Imaging and semi-quantitative elemental analyses of powder samples and precipitates were carried out using a FEI QUANTA FEG 200 SEM–EDS system (Thermo Fisher Scientific). Samples were mounted on conductive carbon tape and examined in low-vacuum mode at an accelerating voltage of 15 kV, without the application of a conductive coating. The SEM was operated with a working distance of 10 mm and a spot size of 3 nm. Standardless EDS was employed for elemental analyses, with a live time of 60 s per spot. Measurements were performed both on isolated larger grains and on homogeneous aggregates of fine particles, with a minimum analysed area of 1 μm^2^.

### Experimental investigation of arsenate sequestration and mimetite Pb_5_(AsO_4_)_3_Cl formation

The experiments were designed to investigate substrate-mediated mimetite formation on Pb-modified zeolite. Zeolite rock from the Bystré deposit (Slovak Republic), supplied by ZeoCem, was used in this study. The zeolite was converted to the sodium-exchanged form and subsequently modified by Pb sorption, resulting in a Pb loading of approximately 70 g Pb/kg zeolite. As the preparation procedure has been described previously (Stępień et al., 2025), only a brief summary is provided here, while the full protocol is included in the Supplementary Material.

Experiments with Pb supplied solely in solution were not performed, as the focus here was on reactions occurring at the mineral-fluid interface. Previous studies have extensively explored bulk solution pathways (e.g. Bajda, 2010; Bajda et al., 2007; Comba, 1987; Flis et al., 2011), and the present work specifically addresses heterogeneous, substrate-mediated processes.

This study focused exclusively on the immobilisation of pentavalent arsenic [As(V)]. Trivalent arsenic [As(III)] was not considered, and no analyses were performed for As(III), as its oxidation and removal were outside the scope of this work. Although As(III) is generally considered more toxic and mobile than As(V), this study focuses on As(V) because arsenate is the relevant species for the formation of lead arsenate minerals, including mimetite (Pb_5_(AsO_4_)_3_Cl), which is the targeted immobilization phase investigated herein. Furthermore, under oxic environmental conditions, As(V) oxyanions are commonly the predominant aqueous arsenic species, particularly in many surface water systems (Smedley & Kinniburgh, 2002).

In all experiments, liquid samples were collected using a syringe fitted with a membrane filter (0.22 µm) to separate the aqueous and solid phases prior to analysis. The filtered solutions were analysed according to the procedures described in section “Examination methods and equipment”. Following completion of the experiments, the solid reaction products were recovered, the suspension had been centrifuged to separate the solid phase, rinsed thoroughly with redistilled water, air-dried at 70 °C in an oven, and subsequently characterized using the analytical methods described in section “Examination methods and equipment”.

Unless otherwise stated, all experiments were performed independently in duplicate under identical experimental conditions. The reported values represent the mean of the duplicate experiments, and the corresponding standard deviations are provided where applicable. In a limited number of cases, only a single experiment could be performed because of insufficient sample volume or technical limitations.

#### Influence of pH and initial arsenate concentration on mimetite formation

Mimetite formation and subsequent arsenate immobilisation with respect to pH and initial As concentration, was evaluated experimentally using solutions containing 0.1, 1.0, 5.0, and 50 mg As(V)/L, each tested at pH 2, 4, 6, and 8. The pH was adjusted prior to the experiment and was not modified further during the reaction. All experimental solutions contained 20 mg Cl^−^/L (added as NaCl), because chloride ions are required for the formation of mimetite (Pb_5_(AsO_4_)_3_Cl), which is the target phase responsible for arsenic sequestration. In each experiment, 0.5 g of Pb-modified zeolite powder was suspended in 40 mL of arsenate solution and left to react for 168 h on a laboratory orbital shaker. Experiments were conducted in duplicates. Aliquots (4 mL) of the suspension were collected after 1 h, 24 h, and 168 h.

A control experiment was carried out in the absence of AsO_4_^3−^. Pb-modified zeolite was suspended in a chloride solution (20 mg Cl^−^/L as NaCl) for three weeks at initial pH values of 2, 4, 6, and 8. Aliquots were collected after 7, 14, and 21 days, filtered through a 0.2 μm membrane and analysed. This experiment was designed to evaluate potential Pb release driven by Na^+^/Pb^2+^ ion exchange and to assess whether the presence of chloride alone could promote Pb mobilisation or precipitation (e.g., as PbCl_2_) under the applied experimental conditions.

Furthermore, a reference experiment was conducted reacting 0.5 g of unmodified zeolite with 40 mL of arsenate solution containing 5 mg As(V)/L and 20 mg Cl^−^/L for 168 h. Aliquots were collected after 2 h, 24 h, and 168 h and analysed for arsenic to assess the arsenate removal by zeolite itself.

A stability test was performed on solids resulting from the experiment (spending material) to evaluate the release of both Pb and As from Pb–As–Cl zeolite (Pb-modified zeolite containing mimetite) upon storage. For this, 0.1 g of the material obtained from the reaction at 50 mg As/L and pH 6, was suspended in 40 mL of redistilled water and sampled periodically (after 7, 14 and 21 days). The long-term stability of Pb–As–Cl zeolite was evaluated without pH adjusting (pH of ~ 6), to simulate low ionic strength conditions and assess potential Pb and As release under mildly acidic environment.

To assess the effects of acidity and ionic strength on mimetite stability, geochemical modelling was performed using PHREEQC (version 3.8.6.17100; Parkhurst and Appelo, 1999) with the [modified llnl2023.dat] thermodynamic database. The solubility product constant (K_sp_) of mimetite (Pb_5_(AsO_4_)_3_Cl) was incorporated into the database based on the values reported by Bajda (2010). Two aqueous systems were simulated—redistilled water (pH 6), and NaCl solution containing 20 mg Cl^−^/L (pH 2 and 6); simulations were performed at 25 °C (Tab. SM 2). The *pe* value used in calculations (*pe* = *4.0*) was not based on direct redox measurements but was selected to represent moderately oxidizing conditions under which As(V) is the dominant arsenic species. This assumption was applied to evaluate arsenic speciation and mineral stability under oxidizing conditions. The modelling outputs included aqueous concentrations of Pb, As, and Cl and arsenate speciation (H_3_AsO_4_, H_2_AsO_4_^−^, HAsO_4_^2−^, AsO_4_^3−^) (Tab. SM 3). These results were used to evaluate mimetite stability, Pb release, and the pH-dependent distribution of arsenate species.

#### Reaction kinetics of arsenate sequestration

Time-resolved arsenate sequestration was quantified by reacting 0.5 g of Pb-modified clinoptilolite with 40 mL of 5 mg As(V)/L and 20 mg Cl^−^/L solution at initial pH 6. The suspension was agitated continuously on a laboratory orbital shaker, with brief pauses only to allow for sample collection. Aliquots were withdrawn at 1, 3, 8, 15, 30, 60, 120, 300 and 1440 minutes.

#### Effect of solid-to-solution ratio on arsenate sequestration

The effect of reactive substrate loading on arsenate immobilisation was tested in experiments of 1 g, 2 g, or 5 g of Pb-modified zeolite powder suspended in 40 mL of solution at two initial arsenate concentrations of 5 mg As(V)/L and 50 mg As(V)/L (in the presence of 20 mg Cl^−^/L). The suspensions were shaken on a laboratory orbital shaker and sampled periodically (at 10, 20, 30, 60 minutes, and 24 hours).

#### Evolution of reactivity during repeated reaction cycles

The effect of repeated reaction cycles on arsenate sequestration was examined by subjecting a single portion of 0.5 g of Pb-modified zeolite to five consecutive 60- minute reactions with 40 mL of 5 mg As(V)/L and 20 mg Cl^−^/L solution (pH 6). Fresh solutions containing 5 mg As(V)/L and 20 mg Cl^−^/L were prepared for every run. After each cycle, the suspension was centrifuged, rinsed three times with redistilled water, air-dried, and reused.

#### Influence of coexisting aqueous ions on mimetite precipitation

To determine potential effect of other ions in solution, As immobilisation experiments were repeated in the presence of selected cations (Mg, Al, K, Ca, Mn, Ni, Cu, Zn, Cd) and anions (F^−^, SO_4_^2−^, PO_4_^3−^). A summary of initial ion concentrations is presented in Tables SM 4 and 5. These ions were selected to represent typical inorganic co-occurring species and to assess their competitive effects and potential interference with arsenic removal mechanisms. Each ion was used separately in the solutions containing arsenate and chloride ions. To evaluate the concentration range, experiments were conducted for each ion using As to cation molar ratios of 1:1, 1:2 and 2:1, corresponding to equimolar conditions, excess ion and excess arsenic, respectively. Based on the high arsenate sequestration and minimal lead release observed in previous experiments, an initial pH of 6 was selected to assess the effectiveness of Pb-modified zeolite in promoting arsenate sequestration via mimetite precipitation under these conditions.

In each experiment, 0.5 g of Pb-modified zeolite was reacted with 40 mL of solution containing 5 mg As(V)/L and 20 mg Cl^−^/L (pH 6) for up to 24 h. Some experiments were also run at higher arsenate concentrations (50 mg/L) to facilitate the detection and observation of newly formed phase using SEM–EDS and XRPD, which was challenging at lower concentrations. Although such high As concentrations are less common in nature, they can occur in highly contaminated environments, such as mining-impacted sites. Therefore, experiments at this level were performed only at a single molar ratio (1:1). In all these experiments, the suspensions mixed on a laboratory orbital shaker, were sampled after 1 h and 24 h.

To evaluate the direct interaction between Pb^2+^, AsO_4_^3−^, PO_4_^3−^, and Cl^−^ ions in the absence of the zeolite framework, an additional zeolite-free control experiment was performed under conditions corresponding to the low-concentration experiments (5 mg As(V)/L, pH 6). For this purpose, arsenate-chloride and phosphate solutions were simultaneously added to the Pb^2+^ solution using a peristaltic pump to ensure controlled co-precipitation conditions. This experiment served as a reference for evaluating the potential formation of mixed mimetite-pyromorphite phases and interpreting mineral formation pathways in the zeolite-based system.

#### Arsenate sequestration in natural waters

Natural waters collected from three arsenic-contaminated sites were used for tests: (i) Bad Wildbad (Vital Therme, Germany; pH 7.59; As(V)=0.14 mg/L), (ii) Złoty Stok (Trująca River, Poland; pH 7.3; As(V)=0.77 mg/L), and (iii) two samples from Upper Mur Valley (Knittelfeld, Austria) (Upper Mur Valley A: pH 7.0; As(V)=0.24 mg/L; Upper Mur Valley B: pH 7.4; As(V)=0.38 mg/L). Similarly to other experiments, a portion of 0.5 g of Pb-modified zeolite was reacted for 24 h with 40 ml of natural water, pH not adjusted, and samples were collected at 1 h and 24 h. Redox potential measurements were performed for the investigated water samples in the laboratory after transport and storage, prior to the immobilisation experiments, using a platinum redox electrode (ELMETRON ERPt-111). The measured redox potentials were within a narrow positive range (approximately + 50 to + 60 mV) and showed no significant differences among the investigated samples. It should be noted that laboratory redox measurements performed after sample transport and storage represent the conditions at the beginning of the experiments and may not fully correspond to the original in situ conditions. Chemical composition of natural waters used is presented in Table SM 6.

## Results and discussion

### Precipitation of mimetite Pb_5_(AsO_4_)Cl on Pb-modified zeolite

**Fig. 1:**
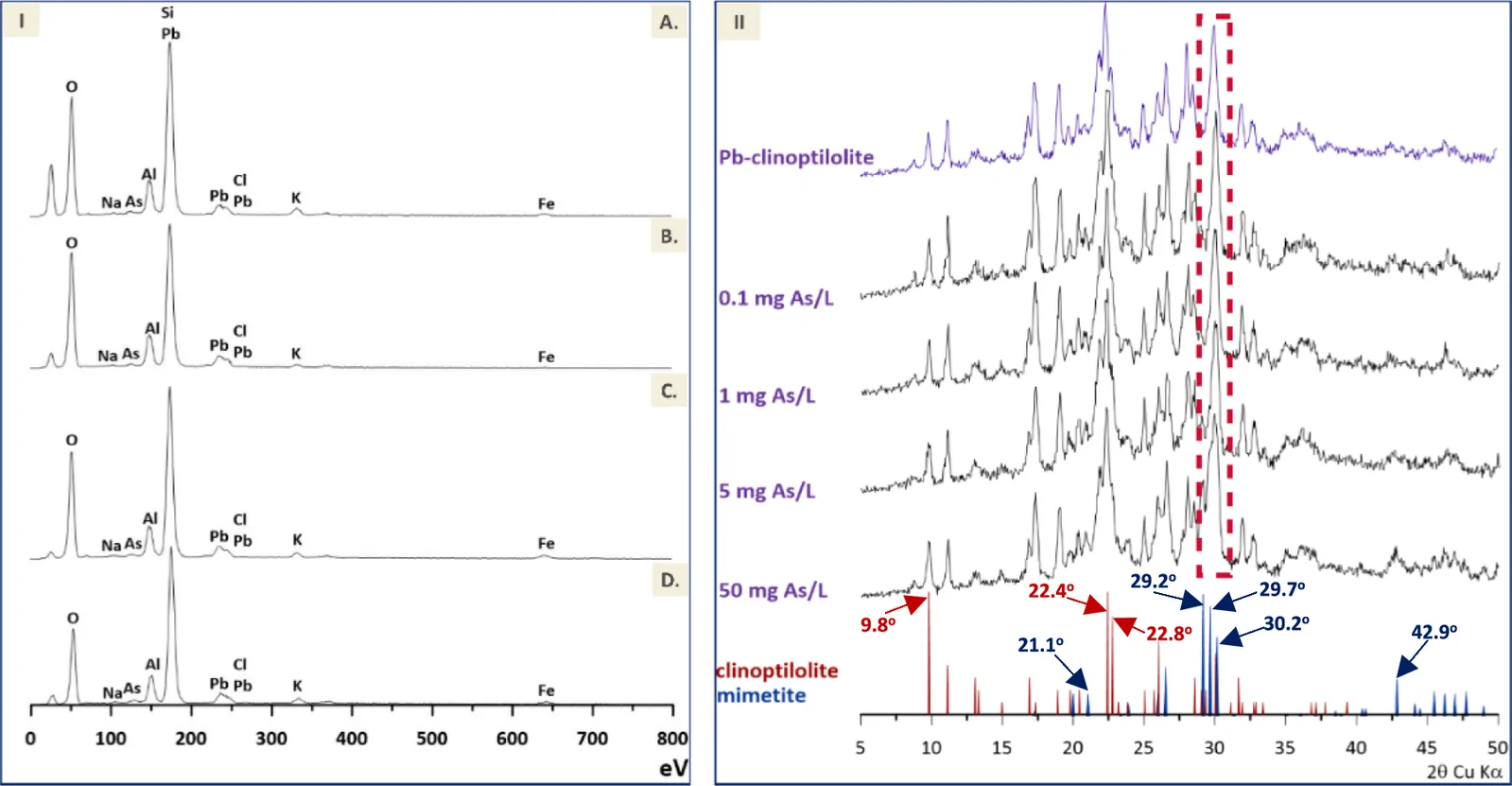
I. Selected EDS spectra confirming the precipitation of lead and chloride arsenates (0.1, 1.0, 5.0, and 50 mg As(V)/L—panels A, B, C, and D, respectively). Spectra were collected from the areas shown in Fig. 2A–D, and acquired by wide-area scans covering the entire SEM fields of view. II. Mimetite Pb_5_(AsO_4_)_3_Cl identified on XRPD patterns (red dashed lines) of Pb-modified zeolite reacted with solutions of various As content at pH 6. Diagnostic reflection peaks and minor peaks used for fingerprinting are indicated by the corresponding 2θ values (clinoptilolite – red colour, mimetite – blue colour)

**Fig. 2:**
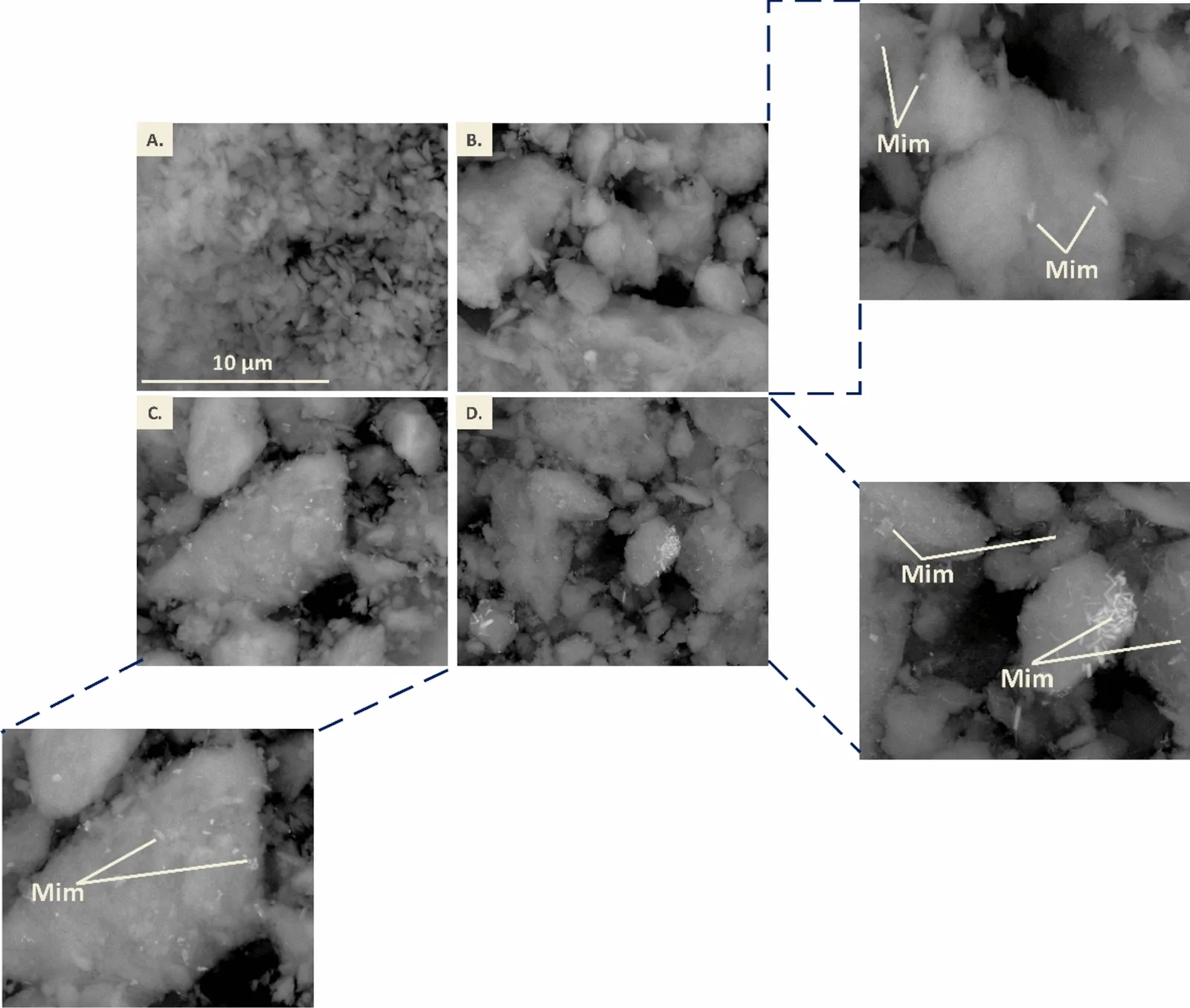
SEM-BSE images of Pb-modified zeolite after reaction with arsenate solutions (**A**–**D**: 0.1, 1, 5, and 50 mg As(V)/L) in the presence of 20 mg Cl^−^/L. Pb-modified zeolite shows a granular, porous structure, while needle-like mimetite (Mim) precipitate formed on the surface, coating the zeolite grains. At 0.1 mg As(V)/L (**A**), mimetite was below SEM detection but confirmed by EDS and As sequestration from solutions. Images shown for initial pH 6; similar behaviour occurred across all tested pH values. The scale bar shown in panel (**A**) applies to all panels (**B**–**D**), which were captured at the same magnification

Arsenate sequestration in the presence of Pb-modified clinoptilolite and dissolved chloride proceeded via the crystallization of a secondary Pb–As–Cl phase identified as mimetite (Pb_5_(AsO_4_)_3_Cl). Its formation was confirmed by XRPD and SEM/EDS (Figs. 1 and 2), which revealed characteristic morphologies, including acicular, platy, and grainy crystals, consistent with previously reported textures and compositions (Stępień et al., 2025).

To assess the role of the zeolite framework itself, unmodified clinoptilolite was tested under identical conditions (0.5 g zeolite in 40 mL of 5 mg/L As solution, contact time up to 168 h). No measurable arsenate uptake was detected, indicating that the zeolite alone did not immobilize arsenate. Arsenate sequestration in the modified system was therefore strongly associated with the availability of Pb^2+^ and the formation of a secondary Pb–As–Cl phase. The absence of measurable arsenate uptake by unmodified clinoptilolite under identical conditions indicates that direct arsenate retention by the zeolite framework alone was not a significant sequestration pathway under the conditions investigated. However, the present experiments did not directly characterize surface properties or arsenate surface species; therefore, a contribution from surface-associated processes cannot be completely excluded. The results are consistent with and extend our previous proof-of-concept study (Stępień et al., 2025), supporting the interpretation that Pb-modified clinoptilolite acts predominantly as a source of Pb^2+^ and a reactive substrate for mineral precipitation.

Pb-modified clinoptilolite consistently triggered mimetite formation across all experiments, supporting a precipitation-controlled mechanism. The newly formed phase was identified qualitatively by XRPD, SEM, and EDS. Theoretical calculations based on the initial arsenate concentrations and assuming complete conversion of removed arsenate into mimetite indicated that the expected mimetite content ranged from approximately 0.0053–2.64 wt.% of the reacted solid phase. Semi-quantitative phase estimation was performed using QUALX. Although mimetite was identified in the reacted samples, its quantitative contribution was very low and close to the detection limit of powder XRD, particularly for samples treated with low initial arsenate concentrations (0.1–1 mg As(V)/L), where mimetite reflections were weak. Therefore, the obtained phase proportions were interpreted qualitatively rather than as absolute mineral abundances. In these cases, SEM and EDS analyses (Figs. 1I, 2; Fig. SM 5 I) were essential for confirming the presence of Pb–As–Cl precipitates.

Textural observations showed that mimetite commonly occurred on or immediately adjacent to the zeolite surface, including partial incrustations on individual grains. Well-formed euhedral crystals were occasionally observed in the surrounding solution, indicating that precipitation was not restricted to the zeolite surface (Fig. 2; Fig. SM 5). The frequent occurrence of surface-associated mimetite is consistent with heterogeneous nucleation at the zeolite-solution interface, although the available observations do not allow the relative contributions of surface-mediated processes and precipitation from the bulk solution to be quantified. These observations support the interpretation that the zeolite provided both a local source of Pb and a surface on which Pb–As–Cl mineral precipitation could occur. Across the investigated pH range (2–8), the crystallization pathway remained broadly consistent, while the extent of mimetite formation depended strongly on both initial arsenate concentration and solution pH. Taken together, these observations suggest that arsenate sequestration was strongly influenced by aqueous geochemical conditions controlling Pb release and the degree of supersaturation with respect to mimetite.

### pH-dependent arsenate immobilisation and Pb release

The extent of arsenate immobilisation in the Pb-modified zeolite system was strongly dependent on solution pH, whereas the effect of initial arsenate concentration was secondary under conditions favourable for mimetite stability. Final pH values remained close to the initial values throughout the experiments, generally within ± 0.3 pH units (Table 1), indicating that the system evolved under relatively stable bulk solution conditions.

**Fig. 3:**
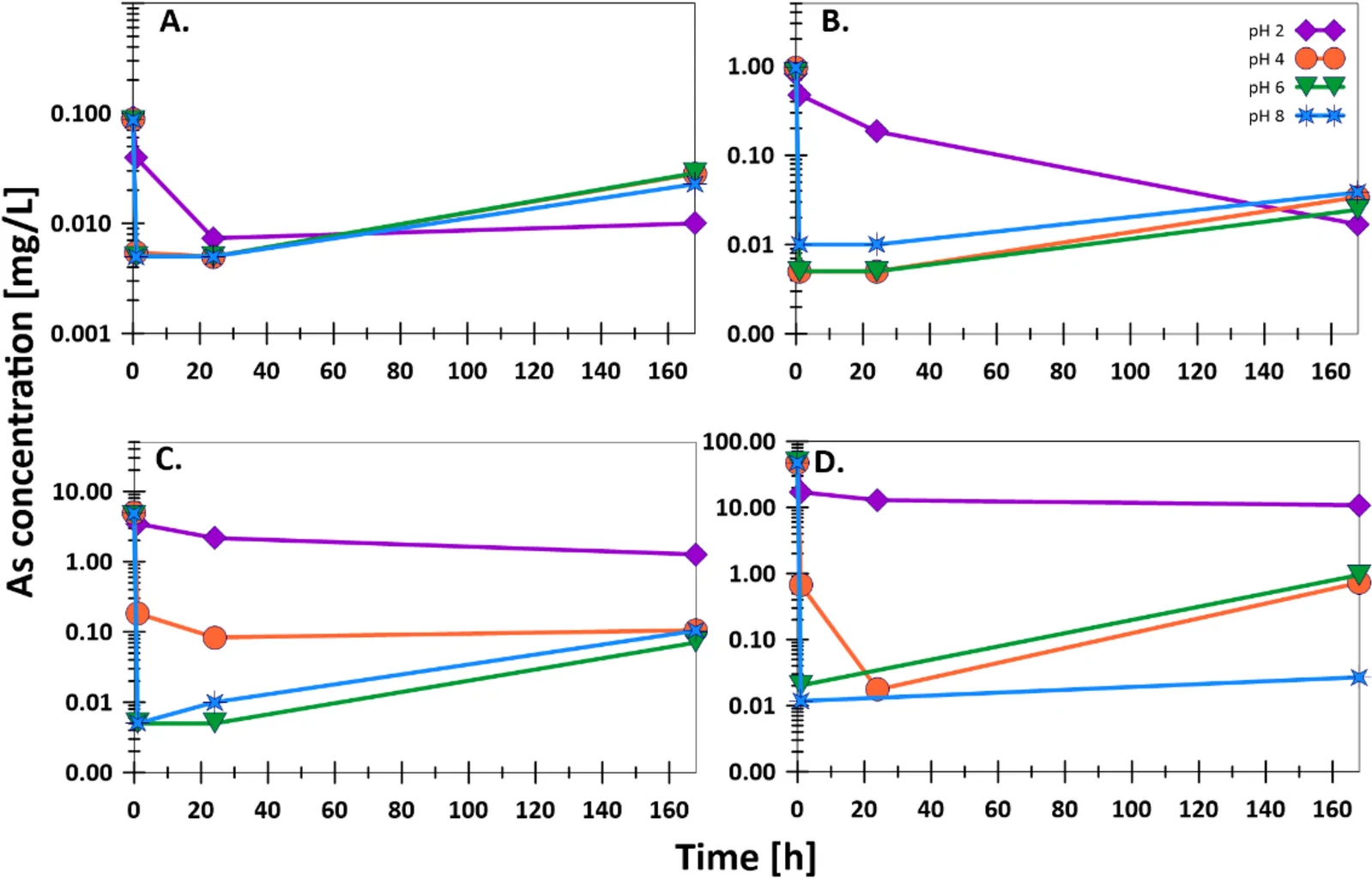
Pb-modified zeolite immobilized arsenate from solution across all initial concentrations (0.1, 1.0, 5.0, and 50 mg As(V)/L—**A**, **B**, **C**, and **D**, respectively) and pH values (2–8). After 1 h, sequestration was lowest at pH 2 (26–65%), while at pH 4–8 it exceeded 94%, reaching 99%. After 24 h, immobilisation further increased, with nearly complete sequestration at pH ≥ 4 and significantly improved at pH 2 (53–92%). After 168 h at pH 4–8 overall As uptake remained high (85–99%). At pH 2, it stabilized in the range of ~ 40–90% depending on the initial concentration

**Table 1:** Initial and final pH of experimental solutions reacted with Pb-modified zeolite for 24 h

Initial As(V) [mg/L]	pH							
	Initial	Final	Initial	Final	Initial	Final	Initial	Final
0.1	2	2.16	4	3.93	6	5.93	8	8.30
1.0		2.13		4.10		6.30		8.30
5.0		2.27		3.91		5.86		7.70
50.0		2.10		3.98		5.75		7.98

Arsenate immobilisation was least effective at pH 2, where residual dissolved As(V) ranged from 0.04 to 17.0 mg/L and sequestration efficiencies were limited to 26–65% (Table 2; Fig. 3). Increasing the pH by only two units produced a marked shift in behaviour: at pH 4, arsenate sequestration exceeded 94%, whereas at pH 6–8 immobilisation was nearly complete, commonly exceeding 99.9% within 1 h. After 24 h, arsenate concentrations decreased further, particularly under acidic conditions, where sequestration increased to 53–92%. At circumneutral to slightly alkaline pH, final dissolved As concentrations were consistently below detection limits.

**Table 2:** Final concentrations of arsenic (mg As(V)/L) along with arsenic sequestration [%], after 1 and 24 h of reaction between Pb-modified zeolite and arsenate solution in the presence of chloride ions (20 mg Cl^−^/L), at arsenic concentrations ranging from 0.1 to 50 mg As(V)/L and pH values between 2 and 8

Initial As(V) [mg/L]	Final As(V) concentration [mg/L]									
	Pb-modified zeolite								Raw zeolite (without modification)	
	pH 2		pH 4		pH 6		pH 8		pH 6	
	1 h	24 h	1 h	24 h	1 h	24 h	1 h	24 h	1 h	24 h
0.1	0.039	0.007	0.005	< 0.005	< 0.005	< 0.005	< 0.005	< 0.005	–	–
1.0	0.476 ± 0.116	0.185 ± 0.076	< 0.005	< 0.005	< 0.005	< 0.005	< 0.01	< 0.01	–	–
5.0	3.464 ± 0.174	2.166 ± 0.222	0.183 ± 0.014	0.083 ± 0.035	< 0.005	< 0.005	< 0.005	< 0.01	5.00	5.00
50.0	17.024 ± 3.582	12.873 ± 1.587	0.668 ± 0.103	0.020	0.020	< 0.01	0.01	< 0.01	–	–

Initial As(V) [mg/L]	As(V) sequestration [%]									
	Pb-modified zeolite								Raw zeolite (without modification)	
	pH 2		pH 4		pH 6		pH 8		pH 6	
	1 h	24 h	1 h	24 h	1 h	24 h	1 h	24 h	1 h	24 h
0.1	55.56	92.22	94.44	99.99	99.99	99.99	99.99	99.99	–	–
1.0	47.11 ± 11.60	79.44 ± 0.08	99.99	99.99	99.99	99.99	99.99	99.99	–	–
5.0	26.14 ± 3.48	53.82 ± 4.44	96.10 ± 0.28	98.23 ± 0.70	99.99	99.99	99.99	99.99	< 0.01	< 0.01
50.0	64.54 ± 7.16	73.18 ± 3.17	98.61 ± 0.21	99.96	99.99	99.99	99.99	99.99	–	–

Values are presented as mean ± standard deviation (n = 2) when duplicate measurements were available. Values below the detection limit are reported as < LOD and standard deviations were not calculated. Single measurements are reported without error estimates

This pronounced pH dependence is consistent with the solubility behaviour of mimetite and the aqueous speciation of arsenate. Under strongly acidic conditions, reduced immobilisation is attributed primarily to the higher solubility of mimetite, which increases equilibrium concentrations of both Pb and As in solution and limits the net extent of secondary phase formation (Bajda, 2010; Huang et al., 2014; Magalhães & Silva, 2003; Turek et al., 2015). In contrast, at pH 4–8, mimetite is substantially more stable, allowing nearly complete transfer of dissolved arsenate into a sparingly soluble Pb–As–Cl phase.

Arsenate speciation further contributes to this behaviour. At low pH, the dominant dissolved species include highly protonated forms, particularly H_3_AsO_4_, which are less effective in driving precipitation with Pb^2+^. At higher pH values, H_2_AsO_4_^−^ and HAsO_4_^2−^ become dominant and more readily participate in the formation of mimetite, thereby favouring arsenate immobilisation over a wide concentration range (Bajda, 2010; Kanel et al., 2023; Patel et al., 2023; Smedley & Kinniburgh, 2002). Consequently, at pH 6 and 8, arsenate sequestration became largely independent of initial As concentration across the investigated range (0.1–50 mg As(V)/L), whereas at pH 2 the extent of immobilisation remained strongly concentration-dependent.

In addition to aqueous arsenate speciation, pH-dependent changes in the surface properties of clinoptilolite may influence arsenate interactions with the reactive substrate. The point of zero charge (PZC) of clinoptilolite depends on its mineral composition, origin, and surface modification (Bish & Ming, 2018; Goldberg, 2002; Xie et al., 2013); therefore, Pb exchange may alter the surface electrochemical properties of the material. Under acidic conditions, protonation of surface sites could favour interactions between the zeolite surface and protonated or partially deprotonated arsenate species, whereas at higher pH values the negatively charged aluminosilicate framework would generally be expected to provide less favourable electrostatic conditions for retention of arsenate oxyanions. However, PZC, zeta potential, or surface-specific spectroscopic measurements were not performed in the present study, and the contribution of surface complexation or other adsorption processes therefore cannot be quantified directly. The negligible arsenate uptake observed for unmodified clinoptilolite under identical conditions indicates that adsorption by the unmodified zeolite alone was not sufficient to account for the observed As removal. Combined with the observed Pb release and identification of mimetite in the reacted material, these results strongly support a precipitation-dominated interpretation of arsenate immobilisation. Surface interactions may nevertheless have contributed locally, for example by providing sites favourable for heterogeneous nucleation or by influencing the local distribution of reactive species at the mineral-fluid interface. Further surface-specific investigations would be required to resolve the contribution of adsorption and interfacial complexation.

The results also indicate that the Pb-modified zeolite system possessed a substantial capacity for arsenate fixation under favourable aqueous conditions. For example, complete sequestration of As(V) from 40 mL of a 50 mg/L solution corresponded to the immobilisation of approximately 2 mg As, equivalent to the formation of roughly 40 mg mimetite. This would require approximately 28 mg Pb, compared with ~ 35 mg Pb initially present on 0.5 g of Pb-modified zeolite, indicating that a large fraction of sorbed Pb remained geochemically reactive toward arsenate under these conditions.

**Fig. 4:**
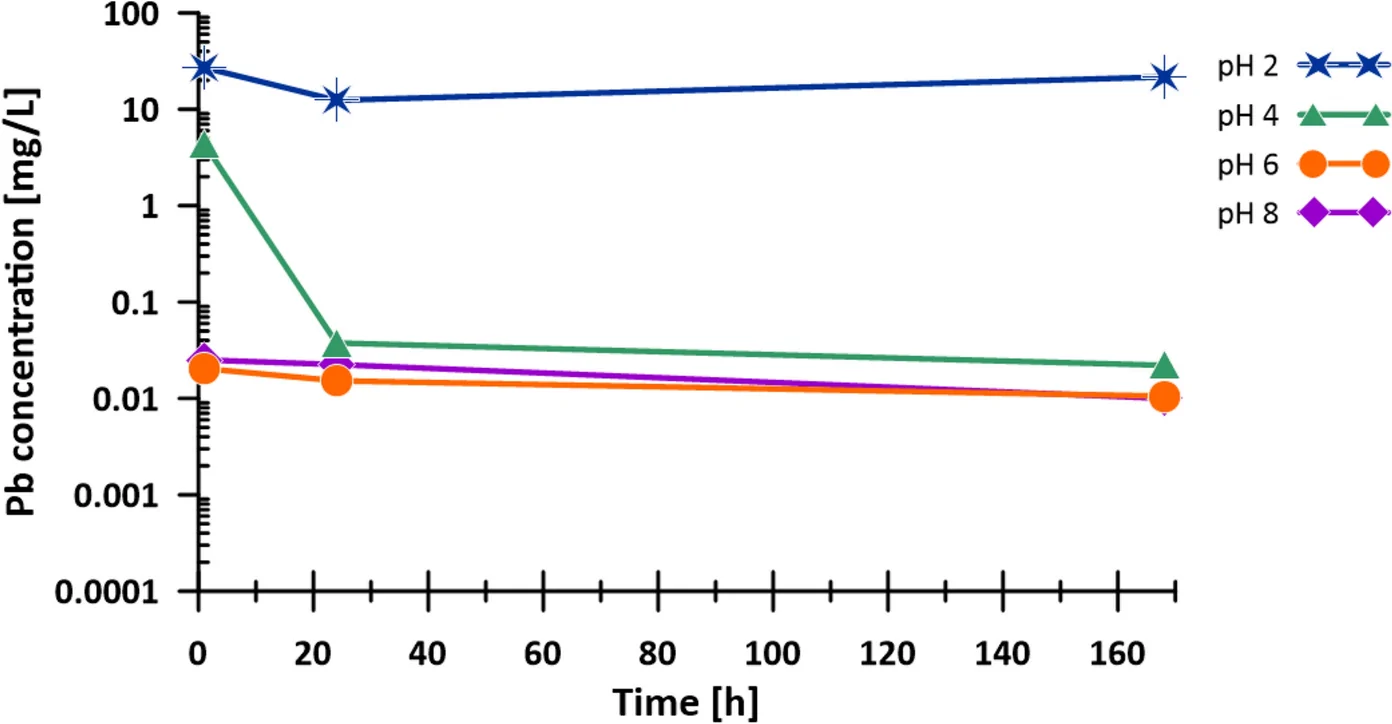
Example of pH-dependent evolution of Pb concentration from after 1 h, 24 h and 168 h of Pb-modified zeolite reaction with a solution containing As (50 mg As(V)/L and 20 mg Cl^−^/L)

The concentration of dissolved Pb after reaction was likewise strongly pH-dependent, reflecting both Pb desorption from the zeolitic substrate and the dissolution–precipitation equilibrium of mimetite. After 1 h, dissolved Pb concentrations ranged from 0.011 to 140.8 mg/L, with the highest values consistently observed at pH 2 (Fig. 4; Table 3). At pH 4–8, Pb concentrations remained low, generally below 0.025 mg/L or at most a few mg/L. Relative to the ~ 35 mg Pb initially sorbed per 0.5 g zeolite, the maximum release at pH 2 corresponded to ~ 16% of sorbed Pb, whereas under circumneutral to alkaline conditions mobilization remained below 1%. With increasing reaction time, dissolved Pb concentrations declined further, particularly under acidic conditions, reaching 1–11% of sorbed Pb after 24 h and 2–10% after 168 h. At pH 4–8, dissolved Pb remained below 1% of total sorbed Pb throughout. SEM and XRPD analyses indicate that clinoptilolite morphology and crystallinity remained largely preserved, even after partial Pb release, although subtle changes below the detection limits of these techniques cannot be excluded. Mimetite was predominantly observed on the zeolite surface, consistent with heterogeneous precipitation, whereas occasional euhedral crystals appeared in the solution phase.

**Table 3:** Final concentrations of lead (Pb) after 1 and 24 h of reaction between Pb-modified zeolite and arsenate solution in the presence of chloride ions (20 mg Cl^−^/L), at arsenic concentrations ranging from 0.1 to 50 mg As(V)/L and pH values between 2 and 8

Initial As(V) [mg/L]	Final Pb concentration [mg/L]											
	pH 2			pH 4			pH 6			pH 8		
	1 h	24 h	168 h	1 h	24 h	168 h	1 h	24 h	168 h	1 h	24 h	168 h
0.1	140.78	98.55	83.66	3.78 ± 0.06	< 0.01	0.49 ± 0.16	0.03 ± 0.01	0.02 ± 0.01	< 0.03	0.46 ± 0.19	0.01	0.02 ± 0.01
1.0	112.92	96.03	32.20	0.04 ± 0.02	0.16 ± 0.07	< 0.03	0.05±0.02	0.03±0.01	< 0.01	0.06 ± 0.02	0.01	0.01
5.0	103.46	72.16	64.30	6.39 ± 0.25	1.60 ± 0.08	0.03 ± 0.01	0.03 ± 0.01	0.18 ± 0.07	0.15 ± 0.06	0.01	0.03 ± 0.01	0.07
50.0	27.03 ± 0.50	12.41 ± 0.22	21.66 ± 0.69	4.39 ± 0.02	0.04 ± 0.02	0.02 ± 0.01	0.02 ± 0.01	0.02 ± 0.01	0.01	0.03 ± 0.01	0.02	< 0.01

Values are presented as mean  ±  standard deviation (n = 2) when duplicate measurements were available. Values below the detection limit are reported as < LOD and standard deviations were not calculated. Single measurements are reported without error estimates

These observations also highlight an important consideration for the environmental application of Pb-modified zeolite. Although the material efficiently immobilized arsenate through the formation of sparingly soluble mimetite under circumneutral conditions, Pb release increased under strongly acidic conditions where mimetite stability decreased. Heightened Pb mobility reflects both enhanced Pb desorption and the higher solubility of mimetite. Consequently, the material may be most suitable for applications in neutral to slightly alkaline waters, whereas use in acidic environments would require careful assessment of Pb mobility and, where appropriate, additional measures to control dissolved Pb. The results therefore demonstrate that the environmental performance of the material depends not only on arsenate removal efficiency but also on maintaining geochemical conditions favourable for secondary Pb mineral stability.

Additional insight into Pb behaviour was obtained from control experiments conducted in 20 mg/L Cl^−^ background solution in the absence of arsenate (Table 4). At pH 2, dissolved Pb reached 48 mg/L, indicating that less than 2 mg Pb (i.e., < 6% of the ~ 35 mg initially present) underwent desorption. Under alkaline conditions (pH 6–8), Pb release was extremely limited, corresponding to less than 0.01% of the Pb associated with the zeolite. These observations indicate that Pb desorption alone was insufficient to account for the behaviour observed in arsenate-bearing systems. Instead, the presence of arsenate promoted Pb mobilization indirectly by removing dissolved Pb^2+^ from solution through mimetite precipitation, thereby sustaining further desorption.

**Table 4:** Final concentrations of Pb released from Pb-modified zeolite during potential Na-Pb ion exchange with NaCl solution (13 mg Na^+^/L, 20 mg Cl^−^/L) at pH 2–8, after 7, 14 and 21 days

Pb [mg/L]				
Time	Initial pH			
	2	4	6	8
7 days	47.96	0.07	0.13	0.11
14 days	48.65	0.16	0.34	0.11
21 days	48.79	0.28	0.21	0.16

Final concentrations of lead (Pb) after 7 to 21 days of reaction between Pb-modified zeolite and chloride solution (20 mg Cl^−^/L), in the absence of arsenate ions, at pH values between 2 and 8. Values are presented as single measurements; error estimates were not calculated for these data

This interpretation is supported by PHREEQC modelling (Tables SM 2 and SM 3), which reproduced the strong pH dependence of the Pb–As–Cl system. At pH 6, equilibrium concentrations of dissolved Pb and As in redistilled water were predicted to be low (0.216 mg/L Pb and 0.047 mg/L As), consistent with the experimentally observed near-complete arsenate immobilisation and minimal Pb release. Under these conditions, arsenate speciation was dominated by H_2_AsO_4_^−^ (~ 93%) with a minor contribution from HAsO_4_^2−^ (~ 7%), both of which are conducive to mimetite precipitation. In contrast, at pH 2, the model predicted substantially higher equilibrium concentrations (77.7 mg/L Pb and 16.9 mg/L As), consistent with partial mimetite dissolution. The aqueous arsenate at this pH was dominated by H_3_AsO_4_ (~ 60%) and H_2_AsO_4_^−^ (~ 34%), with only negligible contributions from more deprotonated species. Together, these calculations support the interpretation that the observed pH dependence arises from the combined effects of secondary mineral stability and arsenate speciation. Although saturation index calculations may provide valuable information on the thermodynamic conditions controlling mimetite precipitation, such modelling is challenging for the present system due to the coupled nature of Pb release from the zeolite and simultaneous mimetite formation. The dissolution and precipitation processes are not independent, and the transient Pb concentrations at the reactive interface cannot be directly determined from bulk solution measurements.

Ionic strength may additionally influence the thermodynamic driving force for mimetite precipitation by modifying the activity coefficients of dissolved species. In the investigated systems, ionic strength varied depending on the initial arsenate concentration and the presence of accompanying ions; however, these changes occurred within a range where mimetite precipitation remained strongly favoured. The activity-based calculations performed using PHREEQC account for these effects and indicate that the observed pH dependence was primarily controlled by mineral stability and arsenate speciation rather than ionic strength alone. Nevertheless, in more saline environments, where activity corrections become more pronounced, ionic strength may represent an additional factor affecting Pb–As–Cl mineral equilibria and should be considered in future assessments.

While ion exchange with Na^+^ may have played a role in Pb desorption, its effect was secondary. This was supported by the control experiment with Na^+^ at concentrations equivalent to those used in the arsenate systems: at pH 6, Pb release remained low and showed no consistent increase in the presence of Na^+^ (Tab. SM 7). The results suggest that Na^+^ alone was unlikely to cause significant Pb mobilization under the experimental conditions. Consequently, Pb concentrations in solution after arsenate sequestration are primarily governed by the dissolution–precipitation equilibrium of mimetite.

Stability experiments performed on the reacted Pb-modified zeolite material (i.e., after mimetite formation) in redistilled water at pH 6 further support the persistence of the sequestration product under circumneutral conditions. Dissolved Pb remained below 0.7 mg/L, while dissolved As(V) continued to decrease over 21 days (Table 5), indicating that no substantial remobilization of As or Pb was observed during the experimental period. However, the 21-day experiment represents only a short-term assessment of product stability and cannot be used to demonstrate long-term environmental persistence.

**Table 5:** Long-term stability of Pb–As–Cl zeolite: final concentrations of arsenic (As) and lead (Pb) measured after 7–21 days of dissolution of mimetite-bearing zeolite in redistilled water at pH = 6

Initial pH = 6		
Time	Pb [mg/L]	As(V) [mg/L]
7 days	0.55	0.32
14 days	0.57	0.14
21 days	0.66	0.08

Values are presented as single measurements; error estimates were not calculated for these data

The low release of As and Pb is consistent with the low solubility and high thermodynamic stability reported for mimetite (Magalhães & Silva, 2003; Bajda, 2010; Solecka et al., 2025), but thermodynamic stability alone does not account for potential kinetic, surface-mediated, or matrix-dependent processes that may influence long-term behaviour. In particular, the stability of the reacted material under fluctuating pH, redox conditions, ionic strength, competing ligands, and continuous-flow conditions remains to be established. Therefore, the present results should be interpreted as evidence of short-term stability under controlled circumneutral conditions rather than as proof of long-term environmental persistence. Longer-duration experiments under environmentally relevant and dynamic conditions will be necessary to evaluate the durability and practical applicability of the material.

Overall, the results are consistent with a coupled desorption-precipitation mechanism operating at and near the mineral-fluid interface. In the absence of arsenate, Pb release from the zeolite remained limited. In the presence of arsenate, the available evidence suggests that desorbed Pb^2+^ was subsequently incorporated into mimetite, which would favour continued Pb release from exchangeable or weakly bound sites on the zeolite by lowering the dissolved Pb concentration. This process can be described schematically by the following reactions, depending on the dominant aqueous arsenate species (Kanel et al., 2023; Stumm & Morgan, 2013):

- At low pH:
  $$5{\mathrm{P}\mathrm{b}}^{2+}+3{\mathrm{H}}_{3}{\mathrm{AsO}}_{4}+{\mathrm{Cl}}^{-}\to {\mathrm{Pb}}_{5}({\mathrm{AsO}}_{4}{)}_{3}\mathrm{C}\mathrm{l}\downarrow +9{\mathrm{H}}^{+}$$
- At intermediate pH:
  $$5{\mathrm{P}\mathrm{b}}^{2+}+3{\mathrm{H}}_{2}{\mathrm{AsO}}_{4}^{-}+{\mathrm{Cl}}^{-}\to {\mathrm{Pb}}_{5}({\mathrm{AsO}}_{4}{)}_{3}\mathrm{C}\mathrm{l}\downarrow +6{\mathrm{H}}^{+}$$
  $$5{\mathrm{P}\mathrm{b}}^{2+}+3{\mathrm{HAsO}}_{4}^{2-}+{\mathrm{Cl}}^{-}\to {\mathrm{Pb}}_{5}({\mathrm{AsO}}_{4}{)}_{3}\mathrm{C}\mathrm{l}\downarrow +3{\mathrm{H}}^{+}$$
- At circumneutral to slightly alkaline pH:
  $$5{\mathrm{P}\mathrm{b}}^{2+}+3{\mathrm{HAsO}}_{4}^{2-}+{\mathrm{Cl}}^{-}\to {\mathrm{Pb}}_{5}({\mathrm{AsO}}_{4}{)}_{3}\mathrm{C}\mathrm{l}\downarrow +3{\mathrm{H}}^{+}$$

These reactions indicate that mimetite formation is intrinsically linked to proton generation under acidic to mildly acidic conditions, which may locally enhance Pb desorption and influence interfacial reaction progress. Mimetite formation occurred predominantly on the zeolite surface, consistent with heterogeneous nucleation, although occasional well-formed euhedral crystals were observed in the solution phase. The occurrence of euhedral crystals in the solution phase demonstrates that precipitation was not restricted to the zeolite surface; however, the available observations do not allow the relative contributions of surface-associated and bulk-solution precipitation to be quantified. The results also showed that arsenate sequestration by Pb-modified clinoptilolite depended on solution pH and initial arsenate concentration. Under circumneutral to slightly alkaline conditions, the low dissolved concentrations of Pb and As, together with the identification of mimetite on the reacted zeolite, are consistent with efficient incorporation of these elements into the newly formed Pb–As–Cl phase, whereas acidic conditions resulted in higher dissolved Pb and As concentrations, indicating greater mobility under these conditions.

### Temporal evolution of arsenate immobilisation and Pb release

At pH 6, arsenate immobilisation in the Pb-modified zeolite system proceeded rapidly (Table 6), consistent with rapid removal under conditions favourable for mimetite stability. A pronounced decrease in dissolved As(V) concentration occurred within the first 3 min, for example from 5 to 0.64 mg/L, corresponding to approximately 88% sequestration. Arsenic decreased further to 0.07 mg/L after 8 min and fell below the analytical detection limit (0.02 mg/L) after 24 h. This rapid decline is consistent with previous observations that mimetite precipitation can proceed on short timescales once appropriate aqueous conditions and reactant availability are established (Bajda et al., 2007).

**Table 6:** Temporal evolution of arsenate immobilisation and lead release: final concentrations of arsenic (As) and lead (Pb) measured after 1–1440 min of reaction between Pb-modified zeolite and arsenate solution in the presence of chloride ions (20 mg Cl^−^/L), at arsenic concentration 5 mg As(V)/L and pH = 6

Time [min]	As(V) [mg/L]	Pb [mg/L]
0	5.51	–
1	3.77 ± 0.01	0.12 ± 0.05
3	0.64 ± 0.01	0.14 ± 0.05
8	0.07 ± 0.01	0.01 ± 0.01
15	0.09 ± 0.01	0.12 ± 0.05
30	0.07 ± 0.01	0.01 ± 0.01
60	0.03 ± 0.01	0.17 ± 0.05
120	0.03 ± 0.01	0.16 ± 0.01
300	0.02 ± 0.01	0.03 ± 0.02
1440	< 0.02	0.15 ± 0.01

Values are presented as mean  ±  standard deviation (n = 2) when duplicate measurements were available. Values below the detection limit are reported as < LOD and standard deviations were not calculated. Single measurements are reported without error estimates

The observed kinetics are consistent with the rapid development of conditions favourable for local supersaturation with respect to mimetite. Initial Pb release from the zeolitic substrate was likely followed by reaction with dissolved arsenate and chloride, resulting in the formation of a low-solubility Pb–As–Cl phase. The rapid appearance of mimetite in the reacted material, together with the temporal decrease in dissolved As, supports the interpretation that precipitation contributed substantially to the observed arsenate immobilisation.

Dissolved Pb concentrations remained relatively low (0.01–0.16 mg Pb/L) throughout most of the experiment. This behaviour is consistent with rapid consumption of released Pb^2+^ by secondary mineral formation and/or other Pb-retention processes, preventing substantial accumulation of Pb in solution. In combination with the identification of mimetite, the data support a system in which dissolved Pb was controlled by the coupled processes of desorption from the substrate and incorporation into the newly formed Pb–As–Cl phase.

Overall, the kinetic data indicate that under circumneutral conditions the Pb-modified zeolite system evolved rapidly toward low dissolved As concentrations through fast mimetite precipitation, while maintaining relatively low dissolved Pb through continuous incorporation into the secondary mineral phase. The temporal evolution of dissolved As and Pb, together with the mineralogical identification of mimetite in the reacted material, supports a precipitation-dominated interpretation involving Pb release from the zeolitic substrate and subsequent formation of a secondary Pb–As–Cl phase.

### Effect of reactive substrate loading on arsenate immobilisation

Within the range of substrate loadings investigated, the extent of arsenate immobilisation was only weakly dependent on the amount of Pb-modified zeolite added to the system (Table 7). Batch experiments were performed in closed systems using 0.5, 1, 2, or 5 mg of Pb-modified zeolite in 40 mL of solution containing 5 or 50 mg As(V)/L at pH 6 in the presence of 20 mg/L Cl^−^.

**Table 7:** Time-dependent variation in arsenic concentration [mg As(V)/L] during reactions of Pb-modified zeolite with arsenate solutions (initial As concentrations of 5 mg As(V)/L or 50 mg As(V)/L), using zeolite dosages of 1, 2, and 5 g

Time [min]	Initial As(V) = 5 mg/L				Initial As(V) = 50 mg/L			
	0.5 g	1 g	2 g	5 g	0.5 g	1 g	2 g	5 g
10	0.13	0.05	0.04	< 0.02	–	0.01	0.03	0.07
20	0.12	< 0.02	< 0.02	0.09	–	< 0.02	0.01	< 0.02
30	0.11	0.04	< 0.02	< 0.02	–	< 0.02	< 0.02	< 0.02
60	< 0.02	0.08	< 0.02	< 0.02	0.02	< 0.02	< 0.02	< 0.02
1440	< 0.02	0.01	< 0.02	0.01	< 0.01	< 0.02	< 0.02	< 0.02

Concentrations were determined at intervals from 10 min up to 24 h. Values are presented as single measurements; error estimates were not calculated for these data

Across all tested loadings, dissolved arsenic concentrations decreased rapidly within the first minutes of reaction and reached values at or below analytical detection limits after 24 h, consistent with the rapid precipitation behaviour observed in the other experiments. Higher substrate loadings produced only a modest acceleration of arsenate uptake during the earliest stage of reaction (first 20 min), but did not substantially alter the final extent of sequestration.

These results indicate that under circumneutral conditions, the reaction was not strongly limited by the absolute amount of Pb-modified zeolite. Instead, the solid/liquid ratio and solution chemistry primarily controlled the kinetics of arsenate immobilisation by regulating Pb availability and secondary phase formation. Even the lowest substrate loading tested was sufficient to promote rapid mimetite formation under the experimental conditions.

### Evolution of reactivity during repeated reaction cycles

Pb-modified clinoptilolite retained its ability to promote arsenate immobilisation and mimetite formation over multiple consecutive reaction cycles (Table 8). The experiment was conducted at pH 6 using a solution containing 5 mg As(V)/L and 20 mg Cl^− ^/L, with the reaction repeated five times for 1 h each. In each cycle, the reacted solution was replaced with fresh solution, while the same Pb-modified zeolite was reused as the reactive substrate.

**Table 8:** Arsenate sequestration on Pb-modified zeolite over repeated reaction cycles, indicating residual As(V) concentrations [mg/L] after each cycle

Cycle	Initial As(V) = 5 mg/L		
	As(V) [mg/L]	As(V) removal [%]	Pb [mg/L]
1	< 0.02	99.99	0.06
2	< 0.02	99.99	0.04
3	< 0.02	99.99	0.03
4	< 0.02	99.99	0.09
5	0.04	99.24	0.03

Values are presented as single measurements; error estimates were not calculated for these data

Arsenate sequestration remained consistently high throughout the experiment, reaching 99.9% during the first four cycles and decreasing only slightly to 99.2% in the fifth cycle. This indicates that a substantial fraction of Pb associated with the zeolite remained geochemically reactive even after repeated arsenate exposure, and that the system continued to support secondary Pb–As–Cl phase formation under circumneutral conditions.

Dissolved Pb concentrations were low during all cycles, indicating that Pb released from the zeolitic substrate continued to be effectively incorporated into newly formed precipitates rather than accumulating in solution. This behaviour is consistent with the persistence of a coupled desorption-precipitation mechanism, in which exchangeable or weakly bound Pb is progressively mobilized and consumed by mimetite formation during successive reactions.

The slight decrease in arsenate immobilisation observed in the fifth cycle likely reflects the gradual depletion of the most reactive Pb fraction available for release and precipitation. Nevertheless, the overall behaviour indicates that the Pb-modified zeolite system did not lose its reactivity abruptly, but instead maintained its capacity for arsenate sequestration over repeated exposure to arsenate-bearing solutions. These results suggest that under favourable aqueous conditions, Pb-modified clinoptilolite can function as a persistent mineralizing substrate, capable of sustaining repeated mimetite precipitation until the reactive Pb reservoir becomes progressively exhausted.

### Influence of coexisting aqueous ions on mimetite precipitation

#### Influence of accompanying cations

Reaction of Pb-modified clinoptilolite with arsenate-bearing solutions in the presence of a range of coexisting cations consistently resulted in the formation of crystalline mimetite, Pb_5_(AsO_4_)_3_Cl, as confirmed by XRPD (Fig. SM 6I). At lower initial arsenate and cation concentrations, the amount of precipitate was locally visible in SEM but too low for XRPD detection, whereas at elevated arsenate concentration (50 mg As(V)/L) characteristic mimetite reflections became clearly discernible. No additional crystalline phases attributable to the presence of competing cations were identified.

SEM and EDS observations (Fig. SM 6II-III) confirmed the presence of Pb, As, and Cl in the newly formed precipitates and showed that mimetite developed predominantly as fine crystalline aggregates coating the zeolite grains. These precipitates occurred mainly as grainy to acicular incrustations, typically < 1 µm in size, closely resembling the morphologies observed in cation-free systems. This indicates that the fundamental crystallization pathway of mimetite remained largely unchanged in the presence of additional dissolved cations. At an initial arsenate concentration of 5 mg/L, coexisting cations did not substantially inhibit arsenate immobilisation.

Residual dissolved As remained very low in all systems, corresponding to sequestration generally exceeding 97% (Table 9). In the presence of Mg, Ca, K, Ni, Cu, Zn, and Cd, dissolved As after 1 h and 24 h was typically ≤ 0.1 mg/L, indicating that mimetite precipitation remained highly effective. Slightly higher residual concentrations were observed in the presence of Al and Mn, but even in these cases arsenate immobilisation remained above 98%. These results indicate that, within the concentration range studied, the geochemical driving force for mimetite precipitation was sufficiently strong to remain largely insensitive to dissolved cation competition.

**Table 9:** Final concentrations of arsenic and lead, after 1 h and 24 h of reaction between Pb-modified zeolite and solutions containing As(V) (5 mg/L), chloride (20 mg Cl^−^/L), and individual co-occurring ions (Mg^2+^, Al^3+^, K^+^, Ca^2+^, Mn^2+^, Ni^2+^, Cu^2+^, Zn^2+^, Cd^2+^)

Initial [As(V)] = 5 mg/L						
Accompanying cation	Final arsenate concentration [mg As(V)/L]					
	As(V)/cation 1:1		As(V)/cation 1:2		As(V)/cation 2:1	
	1 h	24 h	1 h	24 h	1 h	24 h
Mg	0.06 ± 0.04	0.03 ± 0.01	0.10 ± 0.01	0.04 ± 0.02	0.08 ± 0.01	0.08 ± 0.03
Al	0.09 ± 0.01	0.10 ± 0.02	0.09 ± 0.04	0.10 ± 0.05	0.06 ± 0.004	0.05 ± 0.03
K	0.03 ± 0.01	0.06 ± 0.04	0.06 ± 0.02	0.03 ± 0.01	0.06 ± 0.02	0.04 ± 0.02
Ca	0.04 ± 0.02	0.05 ± 0.03	0.06 ± 0.02	0.03 ± 0.01	0.04 ± 0.02	0.05 ± 0.03
Mn	0.06 ± 0.004	0.07 ± 0.004	0.05 ± 0.05	0.08 ± 0.05	0.09 ± 0.01	0.02 ± 0.01
Ni	0.04 ± 0.01	0.07 ± 0.004	0.05 ± 0.05	0.08 ± 0.05	0.05 ± 0.05	0.07 ± 0.05
Cu	0.04 ± 0.01	0.07 ± 0.01	0.04 ± 0.03	0.10 ± 0.05	0.07 ± 0.01	0.04 ± 0.02
Zn	0.04 ± 0.01	0.08 ± 0.02	0.03 ± 0.03	0.06 ± 0.05	0.05 ± 0.004	0.03 ± 0.01
Cd	0.04 ± 0.01	0.05 ± 0.01	0.13 ± 0.04	0.05 ± 0.04	0.02 ± 0.002	0.04 ± 0.02

Accompanying cation	Final Pb concentration [mg/L]					
	As(V)/cation 1:1		As(V)/cation 1:2		As(V)/cation 2:1	
	1 h	24 h	1 h	24 h	1 h	24 h
Mg	0.33 ± 0.02	0.37 ± 0.01	0.33 ± 0.01	0.42 ± 0.02	0.40	0.38
Al	< 0.01	< 0.01	< 0.01	0.03 ± 0.01	< 0.01	< 0.01
K	0.01 ± 0.017	0.10 ± 0.018	0.16 ± 0.02	0.20 ± 0.02	0.26	0.27
Ca	0.27 ± 0.004	0.29 ± 0.01	0.28 ± 0.01	0.30 ± 0.01	0.32	0.36
Mn	0.68 ± 0.02	0.77 ± 0.45	0.15 ± 0.04	0.15 ± 0.04	< 0.01	< 0.01
Ni	0.04 ± 0.01	0.05 ± 0.004	< 0.01	< 0.01	0.28	< 0.01
Cu	0.03 ± 0.02	0.01 ± 0.01	< 0.01	< 0.01	< 0.01	0.09
Zn	< 0.01	< 0.01	< 0.01	< 0.01	< 0.01	< 0.01
Cd	< 0.01	0.05 ± 0.01	< 0.01	0.10 ± 0.06	< 0.01	< 0.01

Cation concentrations were adjusted to achieve molar ratios of 1:1, 1:2, or 2:1 (arsenate relative to cation). Values are presented as mean ± standard deviation (n = 2) when duplicate measurements were available. Values below the detection limit are reported as < LOD and standard deviations were not calculated. Single measurements are reported without error estimates

In terms of Pb release from the Pb-modified zeolite, the effects of other cations varied more markedly (Table 9). The strongest Pb mobilization was observed in the presence of Mn, where dissolved Pb reached 0.68–0.77 mg/L at the 1:1 ratio but was negligible at 2:1, suggesting that the effect was sensitive to the relative availability of arsenate and Mn. K and Ca also promoted measurable Pb release, which increased somewhat with increasing cation input. In contrast, Cu, Cd, Al, and Zn produced little or negligible Pb mobilization. Importantly, even in the most pronounced cases, dissolved Pb remained below 0.8 mg/L, indicating that the overall stability of the Pb-bearing solid assemblage was preserved despite moderate perturbations to solution chemistry.

The behaviour of the coexisting cations themselves revealed that their partitioning into the system followed pathways largely distinct from that of arsenate (Table 10). Mg and Al were removed almost quantitatively, while Cu, Cd, Zn, Ni, and Mn also showed substantial to strong retention depending on molar ratio and reaction time. K, by contrast, exhibited negligible uptake under most conditions.

**Table 10:** Final concentrations of accompanying cations, after 1 h and 24 h of reaction between Pb-modified zeolite and solutions containing arsenate (5 mg As(V)/L), chloride (20 mg/L), and individual co-occurring ions (Mg^2+^, Al^3+^, K^+^, Ca^2+^, Mn^2+^, Ni^2+^, Cu^2+^, Zn^2+^, Cd^2+^)

Initial [As(V)] = 5 mg/L									
Cation	Cation concentration [mg/L]								
	As(V)/cation 1:1			As(V)/cation 1:2			As(V)/cation 2:1		
	Initial	Final 1 h	Final 24 h	Initial	Final 1 h	Final 24 h	Initial	Final 1 h	Final 24 h
Mg	1.50	0.02 ± 0.01	< 0.10	3.00	< 0.10	< 0.10	0.52	< 0.10	< 0.10
Al	1.80	0.03	0.03	2.88	0.03	0.03	0.76	0.02	0.02
K	2.50	2.50	2.51	5.20	5.20	5.22	1.30	1.30	1.29
Ca	2.50	0.54 ± 0.02	0.56 ± 0.02	5.00	0.49 ± 0.05	0.50±0.05	1.03	0.55±0.01	0.63±0.06
Mn	2.95±0.11	< 0.07	< 0.07	7.10±0.22	1.70 ± 0.20	1.46 ± 0.21	1.62 ± 0.08	0.08 ± 0.03	0.18 ± 0.04
Ni	3.31 ± 0.02	0.62 ± 0.27	0.86 ± 0.37	7.71	2.27 ± 0.11	2.57 ± 0.06	2.25 ± 0.02	0.43 ± 0.01	0.63 ± 0.13
Cu	4.16 ± 0.75	0.04 ± 0.02	< 0.03	8.45	0.11 ± 0.06	0.05 ± 0.03	2.05	< 0.03	< 0.03
Zn	4.64 ± 0.14	< 0.40	< 0.40	7.96 ± 0.19	1.27 ± 0.01	1.35 ± 0.21	2.99 ± 0.07	< 0.40	< 0.40
Cd	7.02 ± 0.04	0.66 ± 0.36	0.89 ± 0.03	14.10 ± 0.14	1.27 ± 0.01	1.35 ± 0.21	4.57 ± 0.15	0.03	0.03

Cation concentrations were adjusted to achieve molar ratios of 1:1, 1:2, or 2:1 (arsenate relative to cation). Values are presented as mean  ±  standard deviation (n = 2) when duplicate measurements were available. Values below the detection limit are reported as < LOD and standard deviations were not calculated. Single measurements are reported without error estimates

When experiments were performed at elevated arsenate concentration (50 mg/L As(V)) at a 1:1 molar ratio with the accompanying cations, arsenate was again removed rapidly and almost completely from solution (Table 11), with residual dissolved As(V) typically < 0.1 mg/L after 24 h, corresponding to sequestration efficiencies above 99%. These results indicate that the precipitation pathway remained highly effective even under substantially increased arsenate loading, demonstrating that mimetite could form efficiently at both the lower and elevated arsenate concentrations investigated.

**Table 11:** Final concentrations of arsenic, lead, and accompanying cations, after 1 h and 24 h of reaction between Pb-modified zeolite and solutions containing arsenate (50 mg As(V)/L), chloride (20 mg/L), and individual co-occurring ions (Mg^2+^, Al^3+^, K^+^, Ca^2+^, Mn^2+^, Ni^2+^, Cu^2+^, Zn^2+^, Cd^2+^)

Initial [As(V)] = 50 mg/L						
Cation	As(V)/cation molar ratio 1:1					
	Final As(V) [mg/L]		Final Pb [mg/L]		Final cation [mg/L]	
	1 h	24 h	1 h	24 h	1 h	24 h
Mg	0.04 ± 0.03	0.08 ± 0.05	1.94 ± 1.09	3.23 ± 0.81	4.86 ± 0.01	2.52 ± 1.07
Al	0.05 ± 0.02	0.04 ± 0.02	1.06 ± 0.54	2.56 ± 0.46	0.20	0.20
K	0.05 ± 0.03	0.05 ± 0.03	2.12 ± 0.79	4.40 ± 0.36	8.07	7.87
Ca	0.04 ± 0.02	0.08 ± 0.05	3.86±1.15	4.94 ± 0.63	15.66 ± 0.47	15.86 ± 0.60
Mn	0.04 ± 0.02	0.05 ± 0.03	1.42±0.52	2.84 ± 0.39	14.13 ± 0.16	17.70 ± 1.45
Ni	0.08 ± 0.01	0.05 ± 0.01	3.78	4.12	15.66 ± 0.47	15.86 ± 0.60
Cu	0.09 ± 0.02	0.07 ± 0.03	< 0.01	0.66 ± 0.44	16.98 ± 0.06	16.87 ± 0.07
Zn	0.08 ± 0.05	0.05 ± 0.03	1.61	< 0.01	15.24 ± 0.27	14.66 ± 0.51
Cd	0.02 ± 0.002	0.01 ± 0.002	< 0.01	< 0.01	20.69 ± 0.37	19.88 ± 0.34

Cation concentrations were adjusted to achieve molar ratios of 1:1, 1:2, or 2:1 (arsenate relative to cation). Values are presented as mean ± standard deviation (n = 2) when duplicate measurements were available. Values below the detection limit are reported as < LOD and standard deviations were not calculated. Single measurements are reported without error estimates

Under these conditions, dissolved Pb concentrations initially increased, reflecting transient mobilization from the Pb-modified zeolite and/or partial dissolution before re-equilibration of the Pb–As–Cl system. With time, however, dissolved Pb concentrations decreased, consistent with progressive incorporation of Pb into newly formed Pb–As–Cl precipitates and/or other Pb-retention processes within the reactive assemblage (Table 11). After 24 h, dissolved Pb concentrations were broadly similar in the presence of Mg, Al, K, Ca, Mn, and Ni (approximately 2.5–5 mg/L), whereas lower residual Pb was observed in the presence of Cu (~ 0.7 mg/L), and Pb remained below detection in the Zn- and Cd-bearing systems. This behaviour suggests that although higher arsenate loading temporarily enhanced Pb mobilization, progressive mimetite formation ultimately buffered dissolved Pb concentrations.

The retention behaviour of the coexisting cations at 50 mg/L As(V) remained broadly comparable to that observed at lower arsenate concentrations, although some shifts in uptake efficiency were evident (Table 11). Al^3+^ was again removed almost completely (98.9%), followed by Mg^2+^ (83.2%). The retention of other ions was moderate and relatively similar, with uptake efficiencies of 68.5% for K^+^, and 63.4% for Ca^2+^, 59.4% for Cu^2+^, 71.7% for Cd^2+^, and 69.2% for Zn^2+^, indicating no pronounced preferential immobilization among these ions. Ni^2+^ and Mn^2+^ were retained less efficiently (52.1% and 40.0%, respectively). These results indicate that although absolute uptake varied under higher solute loading, the general partitioning behaviour of the coexisting cations remained consistent with selective retention by the zeolitic substrate and associated secondary processes.

The observations are consistent with the well-established cation selectivity of clinoptilolite, which generally favours divalent transition metals over monovalent alkali and, in many cases, alkaline earth cations. Natural clinoptilolite has long been recognized as exhibiting strong affinity toward ions such as Pb^2+^, Cu^2+^, Cd^2+^, Zn^2+^, Ni^2+^, and Mn^2+^, with uptake behaviour governed by a combination of ionic charge, hydrated radius, hydration enthalpy, and the structural topology of exchange sites (e.g., Grifasi et al., 2025; Minceva et al., 2008; Oter and Akçay, 2007; Ouki and Kavanagh, 1999; Senila & Cadar, 2024; Shaheen et al., 2012; Sprynskyy et al., 2006; Velarde et al., 2023). The uptake sequence observed in the present system broadly follows this established pattern, indicating that the ion-exchange functionality of the zeolite remained active despite the simultaneous presence of arsenate and chloride in solution.

Among the non-transition cations, K^+^ exhibited negligible uptake, consistent with its low charge density and comparatively weak affinity for exchange sites under the experimental conditions. Ca^2+^ displayed more variable behaviour, suggesting a more limited or condition-dependent interaction with the reactive assemblage. Although some degree of exchange is plausible, Ca may also have participated indirectly in the evolving solution chemistry, including possible interactions with arsenate-bearing species or surface precipitates. However, no discrete Ca-bearing arsenate phases were identified by XRPD or SEM–EDS. In contrast, Mg^2+^ was removed efficiently, most likely through a combination of cation exchange and incorporation into poorly ordered or amorphous secondary products. The available XRPD and SEM–EDS data do not allow the contribution of poorly ordered or amorphous Mg-bearing products to be established.

Al^3+^ behaved differently from the divalent cations and was removed almost completely, likely through mechanisms extending beyond simple exchange. Given its strong tendency to hydrolyse, the high Al removal may involve processes beyond simple ion exchange, including hydrolysis and precipitation, surface association, or formation of poorly crystalline Al-bearing products. However, these mechanisms could not be distinguished directly using the methods applied in this study. The absence of identifiable crystalline Al-bearing phases by XRPD and SEM–EDS suggests that any such products, if present, were below the detection limits of these techniques or occurred as poorly crystalline or surface-associated phases.

The divalent transition metals Mn^2+^, Ni^2+^, Zn^2+^, Cd^2+^, and Cu^2+^ were most likely retained primarily through cation exchange, consistent with their generally high affinity for clinoptilolite. The observed differences in their uptake efficiencies likely reflect known differences in hydrated radius, hydrolysis behaviour, and exchange selectivity, as well as the effects of concentration and ionic competition in solution.

Fe is a common constituent of As-bearing waters and can strongly influence arsenate partitioning through adsorption onto Fe(III)-oxyhydroxides and precipitation of Fe-As minerals such as scorodite (FeAsO_4_ · 2H_2_O), these phases may provide important long-term As immobilization pathways due to their relatively low solubility and thermodynamic stability (Drahota & Filippi, 2009; Krause & Ettel, 1989; Smedley & Kinniburgh, 2002). However, it was intentionally excluded from the present study because the primary objective was to investigate arsenate sequestration through mimetite formation. Under the investigated conditions, dissolved Fe is unstable and readily precipitates as Fe-bearing phases or forms Fe(III)-oxyhydroxides capable of efficiently adsorbing and immobilizing arsenate. Preliminary tests confirmed rapid Fe removal from solution, indicating that these competing Fe-controlled processes would occur alongside Pb–As mineralization. Consequently, any decrease in dissolved As could not be unambiguously attributed to mimetite precipitation, making it impossible to distinguish the relative contributions of Fe- and Pb-mediated immobilization pathways. The inclusion of Fe would therefore bias the mechanistic interpretation of the results. It is likely that, in natural waters where both Fe and Pb are present, these processes would act simultaneously and potentially enhance the overall arsenate removal efficiency. However, evaluating such synergistic or competitive interactions was beyond the scope of the present work, which was designed to isolate and quantify the contribution of mimetite formation. Future studies should therefore investigate Fe-containing systems to assess the interplay between Fe–As and Pb–As mineralization pathways under environmentally relevant conditions.

The contrasting behaviour of cations and arsenate is geochemically significant and reflects the fundamentally different modes of interaction available within the system. Because clinoptilolite possesses a negatively charged aluminosilicate framework, arsenate species are not expected to compete directly for exchange positions and therefore cannot be retained through classical cation-exchange mechanisms. Instead, arsenate immobilisation was controlled predominantly by precipitation with Pb released from the zeolitic phase. In contrast, the dissolved cations interacted with the system through pathways including exchange, surface association, hydrolysis, and, where relevant, secondary phase formation, depending on their individual aqueous speciation and reactivity.

Taken together, these results indicate that the presence of coexisting cations did not fundamentally alter the precipitation-driven sequestration of arsenate as mimetite. Rather, the system is best interpreted as a coupled mineral-solution reactive interface in which arsenate immobilisation was governed primarily by secondary Pb–As phase formation, while dissolved cations were simultaneously redistributed through exchange and hydrolysis-related processes. This functional separation between oxyanion immobilisation and cation partitioning provides a useful framework for understanding the stability and persistence of arsenate sequestration in compositionally complex aqueous environments. While additional high-resolution characterization would be required to definitively confirm the speciation and distribution of Ca- and Al-containing products, the primary objective of this study was to investigate substrate-mediated precipitation under variable aqueous conditions rather than to perform nanoscale analysis. The absence of additional crystalline phases in XRPD and SEM/EDS supports the interpretation that no major crystalline competing phases formed, but does not exclude poorly crystalline, amorphous, or surface-associated products. These limitations do not affect the principal conclusion that the presence of the investigated cations did not substantially inhibit arsenate immobilisation or the formation of mimetite.

#### Influence of accompanying anions

**Fig. 5:**
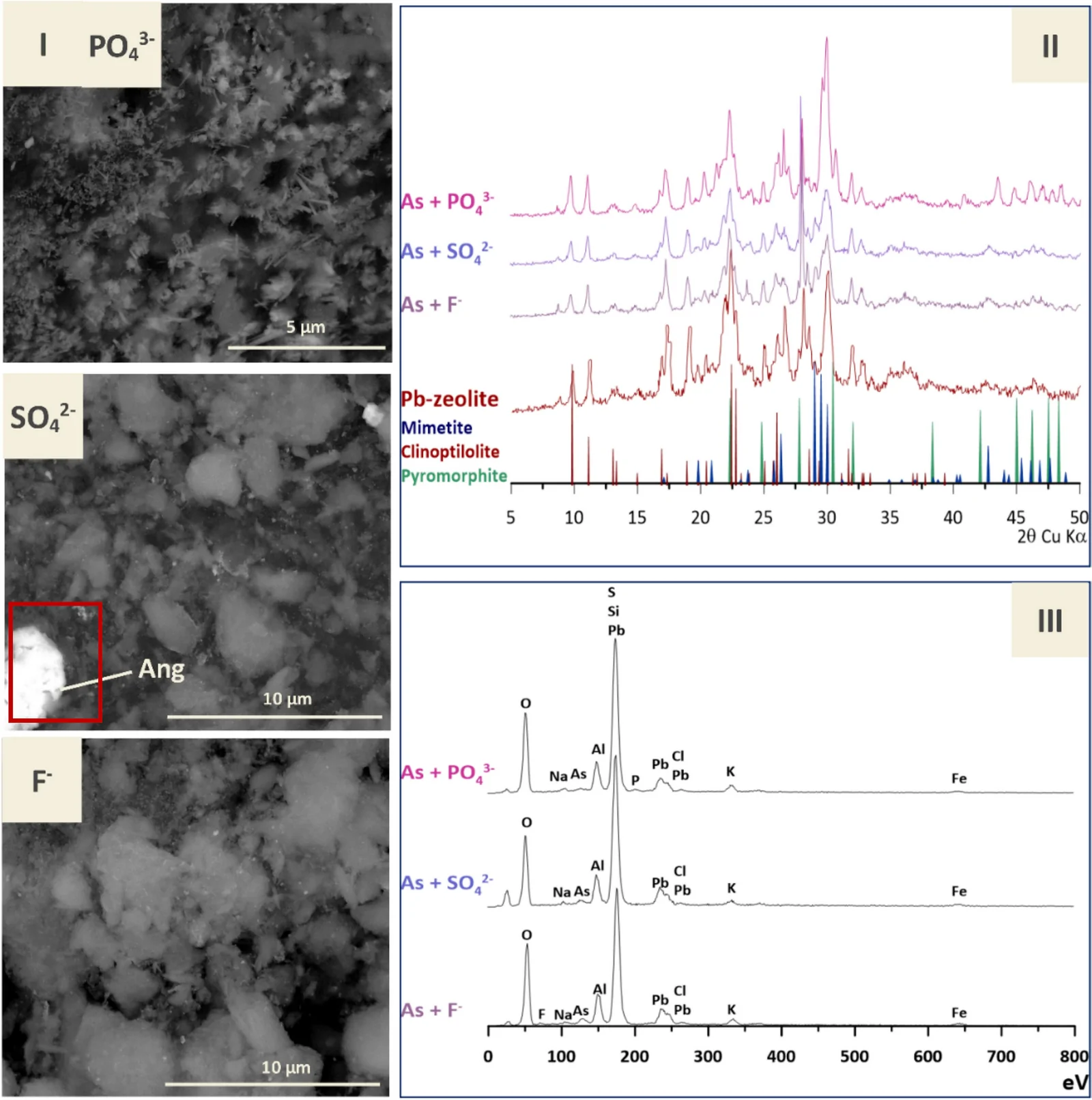
(I) SEM micrographs illustrating surface morphology of Pb-modified zeolite after reaction with As-Cl solution in the presence of different anions, highlighting formation of mimetite incrustations. (II) XRPD patterns showing the formation of mimetite Pb_5_(AsO_4_)_3_Cl after Pb-modified zeolite exposure to arsenate and chloride solution (50 mg As(V)/L and 20 mg Cl^−^/L) for 168 h, with variations depending on the presence of different anions (fluoride F^−^, sulphate SO_4_^2−^ or phosphate PO_4_^3−^, molar ratio 1: (1). (III) SEM–EDS spectra showing elemental composition of precipitates formed, confirming formation of lead chloro-arsenate. In the reaction conducted in the presence of PO_4_^3−^, phosphorus was incorporated into the lead chloro-arsenate phase, indicating the formation of mimetite with admixture of phosphate. EDS spectra were collected from the areas shown in panel I. For PO_4_^3−^ and F^−^ spectra were obtained by wide-area scans covering the entire SEM fields of view, whereas SO_4_^2−^ was analysed by a smaller-area scan of the region marked by the red rectangle

Reaction of Pb-modified zeolite with arsenate-bearing solutions in the presence of accompanying anions led to the formation of mimetite as the principal secondary Pb–As phase (Fig. 5). At lower initial arsenate concentrations, the amount of newly formed material was insufficient for unequivocal XRPD detection; however, SEM–EDS analyses consistently identified Pb, As, and Cl within the precipitates, confirming the formation of lead chloroarsenate. These phases occurred both as surface overgrowths on zeolite grains and as discrete crystals occupying intergranular pore space, typically exhibiting acicular to granular morphologies (Fig. 5I).

In phosphate-bearing systems, phosphorus was additionally detected within the precipitated phases, indicating incorporation of phosphate into the Pb–As–Cl system and suggesting formation of a Pb_5_(AsO_4_,PO_4_)_3_Cl solid solution and/or coexistence of mimetite and pyromorphite. XRPD patterns (Fig. 5II) showed reflections attributable to both end members (characteristic peaks for mimetite—26.3, 29.2, 29.7^°^ 2θ and pyromorphite—30.5, 45.1, 46.3, 47.6, 48.4^o^2θ), while independent synthesis experiments conducted in the absence of zeolite confirmed that mixed Pb chloroarsenate/phosphate phases can form under identical solution conditions (Fig. SM 7C). Because of the close structural and compositional similarity between mimetite and pyromorphite, their distinction by bulk diffraction methods alone remains difficult (Bajda et al., 2011). The formation of a solid solution is plausible, as previous studies have shown that arsenate introduced into phosphate-bearing systems may be incorporated into pyromorphite-type structures, resulting in intermediate members of the pyromorphite-mimetite series rather than discrete end-member phases (Bajda et al., 2011; Campos, 2002; Cao & Ma, 2004; Dhaliwal, 2018; Flis et al., 2011; Impellitteri, 2005; Inegbenebor et al., 1989; Peryea, 1991; Peryea & Kammereck, 1997; Wudarska et al., 2022).

In sulphate-bearing systems, SEM–EDS analyses (Fig. 5I and III) indicated the additional formation of PbSO_4_ (anglesite), occurring as irregular aggregates up to ~ 5 µm in size. Its abundance, however, remained below the detection limit of XRPD.

In fluoride-bearing systems, no secondary phase other than mimetite was identified, although slight decreases in dissolved fluoride concentrations may suggest limited Cl^−^ ↔ F^−^ substitution within the apatite-type structure, consistent with the fluor-mimetite series described by Manecki et al. (2000) and Sordyl et al. (2020).

The assignment of the observed phase relationships should be considered in the context of the analytical limitations of the present study. Changes in dissolved anion concentrations provide evidence for their involvement in the reaction but do not, by themselves, establish the exact molecular or structural mechanism of incorporation. The observed phase assemblages are therefore interpreted as being consistent with the proposed precipitation and substitution pathways rather than as definitive evidence for a single reaction mechanism.

At low initial arsenate concentrations (5 mg As(V)/L), all systems showed extensive arsenate immobilisation (> 97%; Table 12), indicating that the precipitation of Pb–As phases remained the dominant control on dissolved As.

**Table 12:** Final concentrations of arsenic, lead and anions after 1 h to 24 h of reaction between Pb-modified zeolite and solutions containing As(V) (5 mg/L), chloride (20 mg Cl^−^/L), and individual co-occurring ions (F^−^, SO_4_^2−^, PO_4_^3−^)

As(V) concentration [mg/L]						
Molar ratio	1:1		1:2		2:1	
Anion	1 h	24 h	1 h	24 h	1 h	24 h
F^−^	0.05±0.02	0.04±0.02	0.05±0.01	0.04±0.02	0.04	0.05
SO_4_^2−^	0.04±0.03	0.05±0.03	0.06±0.01	0.04±0.03	0.06	0.12
PO_4_^3−^	< 0.005	< 0.005	< 0.005	< 0.005	< 0.005	< 0.005

Pb concentration [mg/L]			
Molar ratio	1:1	1:2	2:1
Anion	24 h		
F^−^	0.16 ± 0.01	0.17 ± 0.01	0.15 ± 0.001
SO_4_^2−^	0.35 ± 0.003	0.29 ± 0.009	0.42 ± 0.04
PO_4_^3−^	0.39 ± 0.04	0.32 ± 0.01	0.35 ± 0.004

Anion concentration [mg/L]						
Molar ratio	1:1		1:2		2:1	
Anion	Initial	Final 24 h	Initial	Final 24 h	Initial	Final 24 h
F^−^	1.20	0.80	2.54	2.20	0.65	0.60
SO_4_^2−^	6.50	6.00	12.00	10.00	3.00	1.00
PO_4_^3−^	6.30	< 0.01	12.00	< 0.01	3.10	< 0.01

Anions concentrations were adjusted to achieve molar ratios of 1:1, 1:2, or 2:1 (As relative to anion). Values are presented as mean ± standard deviation (n = 2) when duplicate measurements were available. Values below the detection limit are reported as < LOD and standard deviations were not calculated. Single measurements are reported without error estimates

Dissolved Pb concentrations after reaction ranged from 0.15 to 0.42 mg Pb/L, with the lowest values observed in fluoride-bearing systems and the highest in sulphate- and phosphate-bearing solutions. These differences indicate that the accompanying anions influenced Pb partitioning during reaction. In phosphate-bearing systems, Pb was distributed between arsenate- and phosphate-bearing apatite-type phases, whereas in sulphate-bearing systems a portion of Pb was diverted toward anglesite formation. The latter is consistent with competition between sulphate and arsenate for dissolved Pb under conditions favouring anglesite formation.

At higher arsenate concentrations (50 mg As(V)/L, 1:1 molar ratio), nearly complete arsenate removal was again observed after 24 h in all systems (≤ 0.02 mg/L residual As; Table 13), confirming that accompanying anions did not suppress Pb-As phase formation. Nevertheless, dissolved Pb concentrations remained strongly dependent on solution composition, reaching up to 5.4 mg Pb/L in sulphate-bearing systems, 1.3 mg Pb/L in fluoride-bearing systems, and 0.67 mg Pb/L in phosphate-bearing systems. These differences are consistent with the relative importance of competing Pb-bearing phases in each system, particularly anglesite in sulphate-rich solutions and mixed apatite-type Pb–As–P phases in phosphate-bearing solutions. Changes in anion concentrations further support these interpretations. Sulphate and fluoride showed only partial decreases in concentration during reaction, whereas phosphate was almost completely removed from solution, indicating its effective incorporation into the precipitated Pb-bearing phase assemblage. This behaviour is consistent with the strong structural compatibility of phosphate and arsenate in apatite-group minerals and supports the possibility of phosphate incorporation into the newly formed Pb-bearing apatite-type phases. The occurrence of mimetite-pyromorphite solid solutions or mixed Pb chloroarsenate/phosphate assemblages is of particular geochemical importance because both end members exhibit very low solubility and high thermodynamic stability across a wide range of aqueous geochemical conditions (Bajda, 2010; Bajda et al., 2007, 2011; Cotter-Howells & Caporn, 1996; Dhaliwal, 2018; Flis et al., 2010, 2011; Ma et al., 1993, 1994, 1995; Magalhães & Silva, 2003; Wudarska et al., 2022; Xu & Schwartz, 1994).

**Table 13:** Final concentrations of arsenic, lead and anions after 1 h to 24 h of reaction between Pb-modified zeolite and solutions containing arsenate (50 mg As(V)/L), chloride (20 mg Cl^−^/L), and individual co-occurring ions (F^−^, SO_4_^2−^, PO_4_^3−^)

As(V) concentration [mg/L]		
Anion	1 h	24 h
F^−^	0.04 ± 0.04	0.02 ± 0.02
SO_4_^2−^	< 0.02	0.02 ± 0.02
PO_4_^3−^	< 0.005	< 0.005

Pb concentration [mg/L]	
Anion	24 h
F^−^	1.31
SO_4_^2−^	5.43
PO_4_^3−^	0.67

Anion concentration [mg/L]		
Anion	Initial	Final 24 h
F^−^	12.00	7.14 ± 0.09
SO_4_^2−^	65.00	9.00 ± 0.41
PO_4_^3−^	63.00	< 0.01

Anions concentrations were adjusted to achieve molar ratios of 1:1 (arsenate relative to anion). Values are presented as mean ± standard deviation (n = 2) when duplicate measurements were available. Values below the detection limit are reported as < LOD and standard deviations were not calculated. Single measurements are reported without error estimates

The observed phase relations are geochemically significant because they show that accompanying anions influence not only dissolved speciation, but also the identity and relative abundance of the secondary Pb-bearing phases formed during reaction. In phosphate-bearing systems, arsenate sequestration proceeded through incorporation into apatite-group phases spanning the pyromorphite-mimetite compositional series. In contrast, sulphate promoted partial redistribution of Pb into anglesite, thereby modifying the partitioning of Pb between arsenate-bearing and non-arsenate-bearing secondary phases. These results indicate that the geochemical evolution of the system is controlled by coupled interactions among ion exchange, Pb release from the zeolite, aqueous ligand competition, and secondary mineral precipitation.From a broader geochemical perspective, the data support the interpretation that arsenate retention in this system is strongly associated with the formation of low-solubility Pb arsenate/chloroarsenate phases, but that the mineralogical pathway appears to depend on the anionic composition of the aqueous phase. This is particularly important in multicomponent natural waters, where coexisting ligands may influence both the speciation of dissolved Pb and the trajectory of secondary mineral formation.

### Mimetite formation in natural waters

**Fig. 6:**
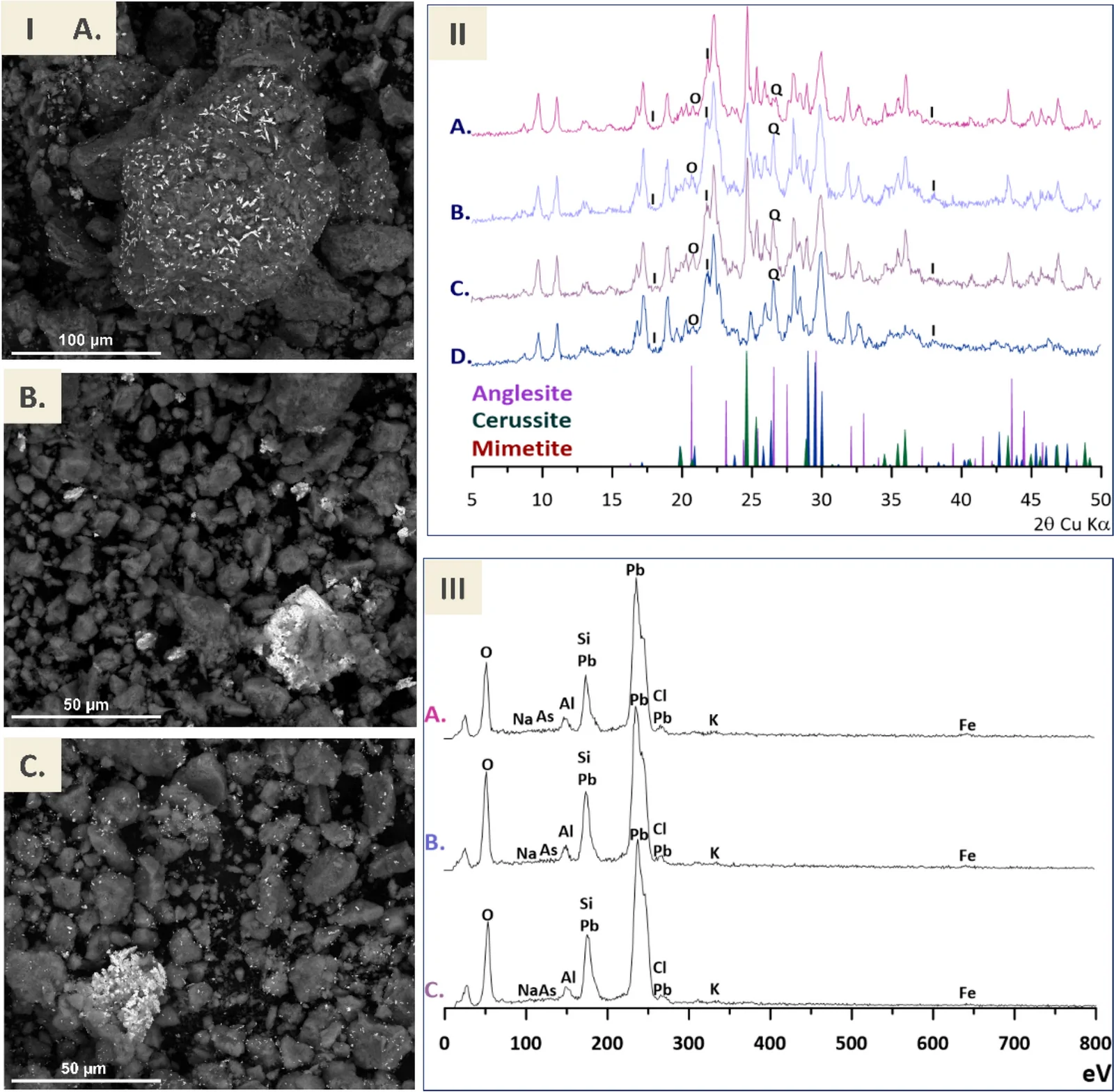
(I) SEM micrographs showing the morphology of Pb-modified zeolite particles after exposure to natural waters containing As: (**A**) Upper Mur Valley, (**B**) Złoty Stok, and (**C**) Bad Wildbad. (II) XRD patterns (A–C) of reacted samples showing the crystallisation of mimetite (Pb_5_(AsO_4_)_3_Cl), along with precipitation of cerussite (PbCO_3_) and anglesite (PbSO_4_). Pattern D. corresponds to Pb-modified zeolite before reaction. (III) EDS spectra from the corresponding areas in (I), confirming the coexistence of Pb, As, and Cl, indicative of mimetite precipitation on the zeolite surface during interaction with natural waters. Spectra were collected from the areas shown in panel I, and acquired by wide-area scans covering the entire SEM fields of view

Reaction of Pb-modified zeolite with natural arsenic-bearing waters resulted in the formation of mimetite as the principal secondary Pb–As phase. XRPD patterns (Fig. 6II) together with SEM–EDS analyses (Fig. 6I and III) confirmed the occurrence of lead chloroarsenate both as surface overgrowths on Pb-modified zeolite grains and as discrete crystals precipitated within intergranular pore space (Fig. 6I). Owing to the more complex aqueous composition of the natural waters, and particularly the presence of HCO_3_^−^ and SO_4_^2−^, additional Pb-bearing phases such as cerussite (PbCO_3_) and/or anglesite (PbSO_4_) were also identified (Fig. 6II), indicating that Pb partitioning in these systems was influenced by competing carbonate- and sulphate-bearing mineralization pathways.

The time-dependent evolution of dissolved arsenic concentrations in the natural water samples is presented in Table 14. The extent of arsenic sequestration varied substantially among the different waters, indicating a strong control of background water chemistry on reaction progress and phase development. In the Bad Wildbad water, residual As(V) concentrations remained relatively high (0.066 mg/L) after both 1 and 24 h, indicating only partial conversion of dissolved arsenic into secondary Pb-As phases. The low As(V) removal observed for Bad Wildbad water may result from its complex geothermal chemistry. This water contains reduced sulphur species (Staatsbad-Wildbad, 2026), which may promote the formation of thioarsenate species that are less favourable for mimetite precipitation than arsenate. Thioarsenates are chemically distinct from arsenate and therefore may not directly participate in mimetite precipitation without prior transformation to arsenate (e.g. Keller et al., 2014). Since mimetite formation involves incorporation of AsO_4_^3−^ into the apatite structure, differences in arsenic speciation may partly explain the lower sequestration efficiency observed for Bad Wildbad water. Additionally, elevated chloride and other dissolved constituents may influence Pb speciation and precipitation pathways. Since arsenic speciation was not directly determined in this study, these effects remain a possible explanation rather than a confirmed mechanism.

**Table 14:** Final concentrations of arsenic (As (V)) along with percentage sequestration, after 1 and 24 h of reaction between Pb-modified zeolite and natural waters samples: Bad Wildbad (pH=7.59), Złoty Stok (pH=7.3), Upper Mur Valley A and Upper Mur Valley B (pH 7.0 and 7.4), at initial arsenic concentrations 0.136, 0.769, 0.243 and 0.379 mg As(V)/L, respectively

Water sample	As (V) concentration [mg/L]			As (V) sequestration [%]		Pb release [mg/L]	
	Initial	1 h	24 h	1 h	24 h	1 h	24 h
Bad Wildbad	0.14 ± 0.001	0.07 ± 0.001	0.07 ± 0.001	51.6% ± 0.71	51.6% ± 0.71	< 0.01	0.54 ± 0.04
Złoty Stok	0.77 ± 0.001	0.07 ± 0.005	0.07 ± 0.001	90.6% ± 0.65	91.2% ± 0.13	0.01±0.07	< 0.01
Upper Mur Valley A	0.24 ± 0.017	< 0.02	0.01±0.007	98.6%	96.8% ± 2.92	< 0.01	< 0.01
Upper Mur Valley B	0.38 ± 0.043	< 0.02	< 0.02	99.9%	99.9%	0.15±0.1	0.38 ± 0.15

Values are presented as mean ± standard deviation (n = 2) when duplicate measurements were available. Values below the detection limit are reported as < LOD and standard deviations were not calculated. Single measurements are reported without error estimates

The highest sequestration efficiencies (> 90%) were observed for surface waters influenced by mining activities (Złoty Stok and Upper Mur Valley samples), which, unlike geothermal waters, are expected to have more oxidizing conditions and therefore a higher proportion of arsenic present as As(V). The Złoty Stok sample showed considerably greater arsenic depletion, with residual concentrations decreasing slightly from 0.072 mg As(V)/L after 1 h to 0.068 mg As(V)/L after 24 h. The Upper Mur Valley waters presented the most extensive decrease in dissolved arsenic: sample A reached very low residual As(V) concentrations after 1 h, whereas sample B showed near-complete arsenic removal (< 0.020 mg As(V)/L) at both sampling times. These differences indicate that the formation and persistence of Pb-As secondary phases in natural waters are highly sensitive to matrix composition. Although mimetite precipitation occurred in all systems, the extent to which dissolved arsenic was transferred into the solid phase varied according to the chemical environment.

In parallel with arsenic removal, dissolved Pb concentrations were monitored to evaluate the aqueous partitioning of Pb during reaction (Table 14). After 1 h, Pb concentrations remained below or close to the detection limit in most systems, indicating that Pb released from the zeolite was initially incorporated efficiently into solid phases. After 24 h, however, differences among waters became more pronounced. Dissolved Pb remained very low in the Złoty Stok and Upper Mur Valley A samples (< 0.01 mg/L), whereas higher Pb concentrations were measured in the Bad Wildbad (0.54 mg/L) and Upper Mur Valley B waters (0.38 mg/L). This behaviour indicates that the long-term distribution of Pb between aqueous and solid reservoirs was strongly dependent on the water chemistry.

The occurrence of cerussite and anglesite in these systems is consistent with carbonate and sulphate influencing the trajectory of secondary mineral formation by providing alternative pathways for Pb partitioning after its release from the zeolite. Under such conditions, a portion of Pb may have been redistributed into non-arsenate Pb-bearing phases, thereby modifying both the extent of mimetite formation and the residual dissolved Pb concentration. This interpretation is consistent with the observations from the synthetic multicomponent systems, where competing anions similarly affected Pb partitioning and the identity of the precipitated secondary phases.

Overall, the results show that the reaction of Pb-modified zeolite with natural arsenic-bearing waters is influenced not only by the intrinsic tendency of Pb and arsenate to form low-solubility secondary phases, but also by the broader geochemical context of the aqueous matrix. In natural waters containing multiple dissolved ligands, arsenic retention and Pb redistribution are coupled to a network of simultaneous processes including ion exchange, aqueous complexation, and competing mineral precipitation. As a result, the mineralogical evolution of the system reflects not a single reaction pathway, but a matrix-dependent sequence of coupled fluid–solid interactions.

These observations extend previous work on Pb-modified zeolite (Stępień et al., 2025), which demonstrated the feasibility of mimetite formation on the reactive substrate under simplified laboratory conditions. The present results show that the same mineralization pathway can operate in chemically complex natural waters, while also revealing that its efficiency and Pb partitioning are controlled by interactions among the reactive substrate, dissolved ligands, and competing secondary mineral phases. This highlights that the environmental applicability of Pb-modified zeolite depends not only on its Pb content but on the ability of the surrounding water chemistry to sustain coupled Pb release and arsenate mineralization.

## 4. Summary and conclusions

This study demonstrates that Pb-modified zeolite acts as a reactive mineral substrate that promotes rapid arsenate sequestration through the formation of mimetite (Pb_5_(AsO_4_)_3_Cl). Building upon our previous proof-of-concept study (Stępień et al., 2025), the present work establishes the geochemical conditions controlling this process and demonstrates that mimetite-based arsenate immobilisation can persist under variable aqueous compositions, including natural arsenic-bearing waters. The process is governed by surface-mediated Pb release from the zeolite, coupled with arsenate complexation and rapid precipitation of secondary Pb-bearing phases as interfacial overgrowths; however, the present data do not allow the contribution of adsorption or other surface-mediated processes to be quantified or completely excluded.References Wang et al. (2025), Krause and Ettel (1989), Solecka et al. (2025) were mentioned in the manuscript; however, this was not included in the reference list. As a rule, all mentioned references should be present in the reference list. Please provide the reference details to be inserted in the reference list.

Arsenate retention in this system is controlled primarily by coupled surface-mediated Pb release and secondary mineral precipitation, rather than direct incorporation into the aluminosilicate framework. The reaction pathway leads to the formation of apatite-group Pb arsenates dominated by mimetite, reflecting the stabilization of a low-solubility mineral phase under the investigated geochemical conditions.

The mineralogical significance of this reaction pathway lies in the formation of mimetite, an apatite-group mineral characterized by very low solubility and high thermodynamic stability over a broad range of geochemical conditions (Bajda, 2010; Magalhães & Silva, 2003; Puzio et al., 2023). In this respect, the Pb–As system differs from other arsenate precipitation pathways, including those involving Ca- or Fe-bearing phases, which may show greater sensitivity to changes in solution composition or pH and may produce less stable secondary products under some conditions (Langmuir et al., 2006; Stefánsson, 2007; Zhu et al., 2006). The observed mineralization therefore represents a distinct geochemical pathway for arsenate incorporation into a sparingly soluble crystalline phase.

The proposed material has potential for treatment of oxidized waters with circumneutral pH, including potentially contaminated groundwater and other aqueous systems with circumneutral pH, where the geochemical conditions favour mimetite stability and minimize Pb release. The 21-day stability experiment provided evidence of limited As and Pb remobilization under controlled circumneutral conditions, but it does not demonstrate long-term environmental persistence of the resulting Pb-bearing phases. Although the low solubility and high thermodynamic stability of mimetite are consistent with potentially favourable long-term retention, the behaviour of the material under prolonged exposure to changing pH, redox conditions, competing ligands, and continuous water flow remains uncertain.

The system behaviour is influenced by aqueous speciation and solution chemistry. The presence of As(V) is essential for efficient sequestration, whereas reduced arsenic species would require prior oxidation (Borho and Wilderer, 1996; Driehaus et al., 1995; Hug & Leupin, 2003; Kim and Nriagu, 2000; Pettine et al., 1999) to participate effectively in mimetite formation. This highlights the importance of redox conditions in governing arsenic mobility and mineral transformation pathways in Pb-bearing systems.

Although Pb is transiently released during reaction, it is rapidly reincorporated into secondary mineral phases, resulting in generally low dissolved Pb concentrations under circumneutral to alkaline conditions. Under acidic conditions, however, higher dissolved Pb concentrations were observed owing to enhanced Pb desorption and the greater solubility of mimetite. Consequently, Pb-modified zeolite is most suitable for remediation under circumneutral conditions, whereas application in acidic environments would require additional measures to control Pb mobility. One possible strategy is the introduction of a phosphate source downstream of the arsenate sequestration stage, promoting precipitation of pyromorphite-group minerals, which are among the least soluble Pb-bearing phases and are widely used for Pb immobilisation in environmental remediation (Bajda et al., 2011; Cotter-Howells & Caporn, 1996; Flis et al., 2011; Laperche, 2000; Li et al., 2022; Ma et al., 1993, 1994, 1995; Qiu et al., 2024; Wang et al., 2013; Xu & Schwartz, 1994). Such an approach could further reduce dissolved Pb concentrations while maintaining efficient arsenate removal.

Competing aqueous species introduce additional controls on phase evolution. While cations are primarily involved in exchange reactions with the zeolitic framework, anions exert a stronger influence on Pb partitioning and secondary mineral stability. In particular, phosphate promotes the formation of apatite-group Pb arsenate/phosphate assemblages, whereas sulphate favours redistribution of Pb into sulfate-bearing phases. These interactions demonstrate that solution composition governs the relative stability of competing Pb-bearing mineral products.

Experiments with natural arsenic-bearing waters confirm that the same coupled mechanism operates under multicomponent geochemical conditions. However, the extent of arsenate sequestration and Pb partitioning is strongly dependent on background water chemistry, which controls both mineral saturation states and the distribution of Pb among competing phases. This highlights the matrix dependence of the reaction and the importance of considering full aqueous speciation when evaluating arsenic mobility in natural systems.

Overall, the system represents a coupled fluid-mineral interface in which Pb-modified zeolite functions both as a transient source of reactive Pb and as a cation-exchanging substrate, while arsenate is transferred from solution into stable secondary Pb-bearing minerals. The geochemical behaviour of the system is controlled by the interplay between Pb release, arsenate speciation, ion exchange processes, and competitive mineral precipitation.

Future work should focus on quantifying the long-term stability of the resulting Pb-bearing mineral assemblages over extended timescales, constraining reaction kinetics under flow-through conditions, characterizing zeolite surface properties and arsenate surface species to resolve the contribution of adsorption and interfacial processes, evaluating the influence of redox variability and multicomponent aqueous chemistry, and assessing integrated treatment strategies, including sequential phosphate addition to immobilize any residual dissolved Pb as pyromorphite-group minerals.

## Supplementary Information

Below is the link to the electronic supplementary material.

Supplementary file1 (DOCX 10108 KB)

## Data Availability

Raw analytical data used in the manuscript will be available on request.

## References

[CR1] Ali, I., Khan, T. A., & Asim, M. (2011). Removal of arsenic from water by electrocoagulation and electrodialysis techniques. *Separation & Purification Reviews,**40*(1), 25–42.

[CR2] Alka, S., Shahir, S., Ibrahim, N., Ndejiko, M. J., Vo, D. V. N., & Abd Manan, F. (2021). Arsenic removal technologies and future trends: A mini review. *Journal of Cleaner Production,**278*, Article 123805.

[CR3] Bajda, T., Szmit, E., & Manecki, M. (2007). Removal of As (V) from solutions by precipitations of mimetite Pb5 (AsO4) 3Cl. Environmental Engineering, pp. 119–124.

[CR4] Bajda, T. (2010). Solubility of mimetite Pb_5_ (AsO_4_) _3_Cl at 5–55 C. *Environmental Chemistry,**7*(3), 268–278.

[CR5] Bajda, T., Mozgawa, W., Manecki, M., & Flis, J. (2011). Vibrational spectroscopic study of mimetite-pyromorphite solid solutions. *Polyhedron,**30*(15), 2479–2485.

[CR6] Banerjee, K., Amy, G. L., Prevost, M., Nour, S., Jekel, M., Gallagher, P. M., & Blumenschein, C. D. (2008). Kinetic and thermodynamic aspects of adsorption of arsenic onto granular ferric hydroxide (GFH). *Water Research,**42*(13), 3371–3378. 10.1016/j.watres.2008.04.019 18538818

[CR7] Bang, S., Korfiatis, G. P., & Meng, X. (2005). Removal of arsenic from water by zero-valent iron. *Journal of Hazardous Materials,**121*(1–3), 61–67. 10.1016/j.jhazmat.2005.01.030 15885407

[CR8] Banning, A. (2021). Geogenic arsenic and uranium in Germany: Large-scale distribution control in sediments and groundwater. *Journal of Hazardous Materials,**405*, Article 124186. 10.1016/j.jhazmat.2020.124186 33127191

[CR9] Battistel, M., Stolze, L., Muniruzzaman, M., & Rolle, M. (2021). Arsenic release and transport during oxidative dissolution of spatially-distributed sulfide minerals. *Journal of Hazardous Materials,**409*, Article 124651. 10.1016/j.jhazmat.2020.124651 33450514

[CR10] Bish, D. L., & Ming, D. W. (Eds.). (2018). *Natural zeolites: Occurrence, properties, applications* (Vol. 45). Walter de Gruyter GmbH & Co KG.

[CR11] Borho, M., & Wilderer, P. (1996). Optimized removal of arsenate (III) by adaptation of oxidation and precipitation processes to the filtration step. *Water Science and Technology,**34*(9), 25–31.

[CR12] Bothe, J. V., & Brown, P. W. (1999). Arsenic immobilization by calcium arsenate formation. *Environmental Science & Technology,**33*(21), 3806–3811.

[CR13] Campos, V. (2002). Arsenic in groundwater affected by phosphate fertilizers at Sao Paulo Brazil. *Environmental Geology,**42*(1), 83–87.

[CR14] Cao, X., & Ma, L. Q. (2004). Effects of compost and phosphate on plant arsenic accumulation from soils near pressure-treated wood. *Environmental Pollution,**132*(3), 435–442. 10.1016/j.envpol.2004.05.019 15325459

[CR15] Chaudhary, M. M., Hussain, S., Du, C., Conway, B. R., & Ghori, M. U. (2024). Arsenic in water: Understanding the chemistry, health implications, quantification and removal strategies. *ChemEngineering,**8*(4), Article 78.

[CR16] Comba, P. G. (1987). Removal of arsenic from process and wastewater solutions (Master’s thesis, Montana Tech of The University of Montana).

[CR17] Cotter-Howells, J., & Caporn, S. (1996). Remediation of contaminated land by formation of heavy metal phosphates. *Applied Geochemistry,**11*(1–2), 335–342.

[CR18] Dhaliwal, I. (2018). Structure and chemistry of pyromorphite, Pb5 (PO4) 3Cl, mimetite, Pb5 (AsO4) 3Cl, vanadinite, Pb5 (VO4) 3Cl, and erythrite, Co3 (AsO4) 2! 8H2O.

[CR19] Drahota, P., & Filippi, M. (2009). Secondary arsenic minerals in the environment: A review. *Environment International,**35*(8), 1243–1255. 10.1016/j.envint.2009.07.004 19665230

[CR20] Driehaus, W., Jekel, M., & Hildebrandt, U. (1998). Granular ferric hydroxide—a new adsorbent for the removal of arsenic from natural water. *Journal of Water Supply: Research and Technology—Aqua,**47*(1), 30–35.

[CR21] Driehaus, W., Seith, R., & Jekel, M. (1995). Oxidation of arsenate (III) with manganese oxides in water treatment. *Water Research,**29*(1), 297–305.

[CR22] Dunkel, F., Niederl, S., Bertrandsson Erlandsson, V., Moser, U., Wagner, S., Irrgeher, J. & Gopon, P. (2025): Raw material potential and environmental impact of historic Cu-Au mine waste at Flatschach, Austria [Poster presentation]. Goldschmidt conference 2025, Prague, Czech Republic. 10.7185/gold2025.28255

[CR23] Flis, J., Borkiewicz, O., Bajda, T., Manecki, M., & Klasa, J. (2010). Synchrotron-based X-ray diffraction of the lead apatite series Pb_10_(PO_4_)_6_Cl_2_-Pb_10_(AsO_4_)_6_Cl_2_. *Synchrotron Radiation,**17*(2), 207–214. 10.1107/S0909049509048705 20157273

[CR24] Flis, J., Manecki, M., & Bajda, T. (2011). Solubility of pyromorphite Pb_5_(PO_4_)_3_Cl-mimetite Pb_5_(AsO_4_)_3_Cl solid solution series. *Geochimica Et Cosmochimica Acta,**75*(7), 1858–1868.

[CR25] Goldberg, S. (2002). Competitive adsorption of arsenate and arsenite on oxides and clay minerals. *Soil Science Society of America Journal,**66*(2), 413–421.

[CR26] Grifasi, N., Ziantoni, B., Fino, D., & Piumetti, M. (2025). Fundamental properties and sustainable applications of the natural zeolite clinoptilolite. *Environmental Science and Pollution Research,**32*(48), 27805–27840. 10.1007/s11356-024-33656-5 PMC12696048 38780851

[CR27] Guan, X. H., Wang, J., & Chusuei, C. C. (2008). Removal of arsenic from water using granular ferric hydroxide: Macroscopic and microscopic studies. *Journal of Hazardous Materials,**156*(1–3), 178–185. 10.1016/j.jhazmat.2007.12.012 18206296

[CR28] Huang, Y. H., Zhu, Z. Q., Zhang, Z. L., Zhu, Y. N., Tan, L. L., Dai, L. Q., & Wei, C. C. (2014). Characterization, dissolution and solubility of mimetite [Pb_5_(AsO_4_)_3_Cl] at 25 C. *Applied Mechanics and Materials,**448*, 15–18.

[CR29] Hug, S. J., & Leupin, O. (2003). Iron-catalyzed oxidation of arsenic (III) by oxygen and by hydrogen peroxide: PH-dependent formation of oxidants in the Fenton reaction. *Environmental Science & Technology,**37*(12), 2734–2742. 10.1021/es026208x 12854713

[CR30] ICDD, PDF-2 Database. (2025). International Centre for Diffraction Data, Newtown Square, PA, USA.

[CR31] Impellitteri, C. A. (2005). Effects of pH and phosphate on metal distribution with emphasis on As speciation and mobilization in soils from a lead smelting site. *Science of the Total Environment,**345*(1–3), 175–190. 10.1016/j.scitotenv.2004.10.024 15919538

[CR32] Inegbenebor, A. I., Thomas, J. H., & Williams, P. A. (1989). The chemical stability of mimetite and distribution coefficients for pyromorphite-mimetite solid-solutions. *Mineralogical Magazine,**53*(371), 363–371.

[CR33] Kanel, S. R., Das, T. K., Varma, R. S., et al. (2023). Arsenic contamination in groundwater: Geochemical basis of treatment technologies. *ACS Environmental Au,**3*, 135–152. 10.1021/acsenvironau.2c00053 PMC10197174 37215436

[CR34] Kanel, S. R., Manning, B., Charlet, L., & Choi, H. (2005). Removal of arsenic (III) from groundwater by nanoscale zero-valent iron. *Environmental Science & Technology,**39*(5), 1291–1298. 10.1021/es048991u 15787369

[CR35] Karczewska, A., Bogda, A., & Krysiak, A. (2007). Arsenic in soils in the areas of former mining and mineral processing in Lower Silesia, southwestern Poland. *Trace Metals and Other Contaminants in the Environment,**9*, 411–440.

[CR37] Keller, N. S., Stefánsson, A., & Sigfússon, B. (2014). Arsenic speciation in natural sulfidic geothermal waters. *Geochimica Et Cosmochimica Acta,**142*, 15–26.

[CR38] Kim, M. J., & Nriagu, J. (2000). Oxidation of arsenite in groundwater using ozone and oxygen. *Science of the Total Environment,**247*(1), 71–79. 10.1016/s0048-9697(99)00470-2 10721144

[CR39] Kleszczewska-Zębala, A., Manecki, M., Bajda, T., Rakovan, J., & Borkiewicz, O. J. (2016). Mimetite formation from goethite-adsorbed ions. *Microscopy and Microanalysis,**22*(3), 698–705. 10.1017/S1431927616000829 27329315

[CR40] Krause, E., & Ettel, V. A. (1989). Solubilities and stabilities of ferric arsenate compounds. *Hydrometallurgy,**22*(3), 311–337.

[CR41] Lackovic, J. A., Nikolaidis, N. P., & Dobbs, G. M. (2000). Inorganic arsenic removal by zero-valent iron. *Environmental Engineering Science,**17*(1), 29–39.

[CR42] Langmuir, D., Mahoney, J., & Rowson, J. (2006). Solubility products of amorphous ferric arsenate and crystalline scorodite (FeAsO4· 2H2O) and their application to arsenic behaviour in buried mine tailings. *Geochimica Et Cosmochimica Acta,**70*(12), 2942–2956.

[CR43] Laperche, V. (2000). Immobilisation of lead by in situ formation of lead phosphates in soils. Environmental Restoration of Metals-Contaminated Soils/Ed. IK Iskandar, 61–75.

[CR44] Lenoble, V., Deluchat, V., Serpaud, B., & Bollinger, J. C. (2003). Arsenite oxidation and arsenate determination by the molybdene blue method. *Talanta,**61*(3), 267–276. 10.1016/S0039-9140(03)00274-1 18969186

[CR45] Li, J., Tian, X., Bai, R., Xiao, X., Yang, F., & Zhao, F. (2022). Transforming cerussite to pyromorphite by immobilising Pb (II) using hydroxyapatite and *Pseudomonas rhodesiae*. *Chemosphere,**287*, Article 132235. 10.1016/j.chemosphere.2021.132235 34826926

[CR47] Long, H., Zheng, Y. J., Peng, Y. L., Jin, G. Z., Deng, W. H., & Zhang, S. C. (2019a). Comparison of arsenic (V) removal with different lead-containing substances and process optimization in aqueous chloride solution. *Hydrometallurgy,**183*, 199–206.

[CR48] Long, H., Zheng, Y. J., Peng, Y. L., Jin, G. Z., Deng, W. H., & Zhang, S. C. (2019b). Study on arsenic removal in aqueous chloride solution with lead oxide. *International Journal of Environmental Science and Technology,**16*(11), 6999–7010.

[CR49] Lu, M., Sun, L., Li, Q., Jiang, H., & Yin, S. (2019). Study on Arsenic Removal by Scorodite. In IOP Conference Series: Earth and Environmental Science (Vol. 218, No. 1, p. 012102). IOP Publishing.

[CR50] Ma, Q. Y., Logan, T. J., & Traina, S. J. (1995). Lead immobilisation from aqueous solutions and contaminated soils using phosphate rocks. *Environmental Science & Technology,**29*(4), 1118–1126. 10.1021/es00004a034 22176421

[CR51] Ma, Q. Y., Traina, S. J., Logan, T. J., & Ryan, J. A. (1993). In situ lead immobilisation by apatite. *Environmental Science & Technology,**27*(9), 1803–1810.

[CR52] Ma, Q. Y., Traina, S. J., Logan, T. J., & Ryan, J. A. (1994). Effects of aqueous Al, Cd, Cu, Fe (II), Ni, and Zn on Pb immobilisation by hydroxyapatite. *Environmental Science & Technology,**28*(7), 1219–1228. 10.1021/es00056a007 22176311

[CR53] Magalhães, M. C. F. (2002). Arsenic. An environmental problem limited by solubility. *Pure and Applied Chemistry,**74*(10), 1843–1850.

[CR54] Magalhães, M. C. F., & Silva, M. C. M. (2003). Stability of lead (II) arsenates. *Monatshefte Fuer Chemie/chemical Monthly,**134*(5), 735–743.

[CR55] Mandal, B. K., & Suzuki, K. T. (2002). Arsenic round the world: A review. *Talanta,**58*(1), 201–235. 18968746

[CR56] Manecki, M., Maurice, P. A., & Traina, S. J. (2000). Uptake of aqueous Pb by Cl-, F-, and OH- apatites: Mineralogic evidence for nucleation mechanisms. *American Mineralogist,**85*(7–8), 932–942.

[CR57] Marciniak, H., Diduszko, R., & Kozak, M. (2006). XRAYAN Program do Rentgenowskiej Analizy Fazowej, Wersja 4.0.1, Koma.

[CR58] Markl, G., Marks, M. A., Holzäpfel, J., & Wenzel, T. (2014). Major, minor, and trace element composition of pyromorphite-group minerals as recorder of supergene weathering processes from the Schwarzwald mining district, SW Germany. *American Mineralogist,**99*(5–6), 1133–1146.

[CR59] Marszałek, H., & Wąsik, M. (2000). Influence of arsenic-bearing gold deposits on water quality in Zloty Stok mining area (SW Poland). *Environmental Geology,**39*(8), 888–892.

[CR60] Martínez-Villegas, N., Briones-Gallardo, R., Ramos-Leal, J. A., Avalos-Borja, M., Castañón-Sandoval, A. D., Razo-Flores, E., & Villalobos, M. (2013). Arsenic mobility controlled by solid calcium arsenates: A case study in Mexico showcasing a potentially widespread environmental problem. *Environmental Pollution,**176*, 114–122. 10.1016/j.envpol.2012.12.025 23416746

[CR61] Minceva, M., Fajgar, R., Markovska, L., & Meshko, V. (2008). Comparative study of Zn^2+^, Cd^2+^, and Pb^2+^ removal from water solution using natural clinoptilolitic zeolite and commercial granulated activated carbon. Equilibrium of adsorption. *Separation Science and Technology,**43*(8), 2117–2143.

[CR62] MohanPittman, D. C. U., Jr. (2007). Arsenic removal from water/wastewater using adsorbents—A critical review. *Journal of Hazardous Materials,**142*(1–2), 1–53. 10.1016/j.jhazmat.2007.01.006 17324507

[CR63] Mojiri, A., Razmi, E., KarimiDermani, B., Rezania, S., Kasmuri, N., Vakili, M., & Farraji, H. (2024). Adsorption methods for arsenic removal in water bodies: A critical evaluation of effectiveness and limitations. *Frontiers in Water,**6*, Article 1301648.

[CR64] Moon, D. H., Dermatas, D., & Menounou, N. (2004). Arsenic immobilization by calcium–arsenic precipitates in lime treated soils. *Science of the Total Environment,**330*(1–3), 171–185. 10.1016/j.scitotenv.2004.03.016 15325167

[CR66] Nazari, A. M., Radzinski, R., & Ghahreman, A. (2017). Review of arsenic metallurgy: Treatment of arsenical minerals and the immobilisation of arsenic. *Hydrometallurgy,**174*, 258–281.

[CR67] Nicomel, N. R., Leus, K., Folens, K., Van Der Voort, P., & Du Laing, G. (2016). Technologies for arsenic removal from water: Current status and future perspectives. *International Journal of Environmental Research and Public Health,**13*(1), 62. 10.3390/ijerph13010062 PMC4730453 26703687

[CR68] Oter, O., & Akcay, H. (2007). Use of natural clinoptilolite to improve water quality: Sorption and selectivity studies of lead (II), copper (II), zinc (II), and nickel (II). *Water Environment Research,**79*(3), 329–335. 10.2175/106143006x111880 17469665

[CR69] Ouki, S. K., & Kavannagh, M. (1999). Treatment of metals-contaminated wastewaters by use of natural zeolites. *Water Science and Technology,**39*(10–11), 115–122.

[CR70] Parkhurst, D. L., & Appelo, C. A. J. (1999). User’s guide to PHREEQC (Version 2): A computer program for speciation, batch-reaction, one-dimensional transport, and inverse geochemical calculations. *Water-Resources Investigations Report,**99*(4259), 312.

[CR71] Patel, K. S., Pandey, P. K., Martín-Ramos, P., Corns, W. T., Varol, S., Bhattacharya, P., & Zhu, Y. (2023). A review on arsenic in the environment: Contamination, mobility, sources, and exposure. *RSC Advances,**13*(13), 8803–8821. 10.1039/d3ra00789h PMC10020839 36936841

[CR72] Peryea, F. J., & Kammereck, R. (1997). Phosphate-enhanced movement of arsenic out of lead arsenate-contaminated topsoil and through uncontaminated subsoil. *Water, Air, and Soil Pollution,**93*(1), 243–254.

[CR73] Pettine, M., Campanella, L., & Millero, F. J. (1999). Arsenite oxidation by H_2_O_2_ in aqueous solutions. *Geochimica Et Cosmochimica Acta,**63*(18), 2727–2735.

[CR74] Plinninger, R. J., & Thuro, K. (1998). Die geologischen Verhältnisse beim Vortrieb des Meisterntunnels Bad Wildbad/Nordschwarzwald. Z. dt. geol Ges., Hannover (in Drucklegung).

[CR75] Podgorski, J., & Berg, M. (2020). Global threat of arsenic in groundwater. *Science,**368*(6493), 845–850. 10.1126/science.aba1510 32439786

[CR76] Puzio, B., Zhang, L., Szymanowski, J. E., Burns, P. C., & Manecki, M. (2023). Thermodynamic characterization of synthetic lead-arsenate apatites with different halogen substitutions. *American Mineralogist: Journal of Earth and Planetary Materials,**108*(4), 675–685.

[CR77] Qiu, L., Yan, C., Munir, T., Wang, Y., Wang, E., Li, R., Wu, X., Huang, Y., & Li, B. (2024). Comparing struvite, K-struvite and hydroxyapatite for the remediation of lead and cadmium contaminated soil. *Sustainable Horizons,**10*, Article 100084.

[CR78] Roy, M., Giri, A. K., Dutta, S., & Mukherjee, P. (2015). Integrated phytobial remediation for sustainable management of arsenic in soil and water. *Environment International,**75*, 180–198. 10.1016/j.envint.2014.11.010 25481297

[CR79] Senila, M., & Cadar, O. (2024). Modification of natural zeolites and their applications for heavy metal removal from polluted environments: Challenges, recent advances, and perspectives. *Heliyon*. 10.1016/j.heliyon.2024.e25303 PMC10862511 38352776

[CR80] Shaheen, S. M., Derbalah, A. S., & Moghanm, F. S. (2012). Removal of heavy metals from aqueous solution by zeolite in competitive sorption system. *International Journal of Environmental Science and Development,**3*(4), 362–367.

[CR81] Shih, M. C. (2005). An overview of arsenic removal by pressure-drivenmembrane processes. *Desalination,**172*(1), 85–97.

[CR82] Siuda, R., & Macioch, A. (2018). Secondary arsenic minerals from the Złoty Stok As–Au abandoned mine (SW Poland). *Geological Quarterly,**62*(4), 925–940.

[CR83] Smedley, P. L., & Kinniburgh, D. G. (2002). A review of the source, behaviour and distribution of arsenic in natural waters. *Applied Geochemistry,**17*(5), 517–568.

[CR85] Sordyl, J., Puzio, B., Manecki, M., Borkiewicz, O., Topolska, J., & Zelek-Pogudz, S. (2020). Structural assessment of fluorine, chlorine, bromine, iodine, and hydroxide substitutions in lead arsenate apatites (mimetites)–Pb_5_(AsO_4_)_3_X. *Minerals,**10*(6), 494.

[CR120] Solecka, U., Puzio, B., Kersten, M., Topolska, J., Manecki, M., & Bajda, T. (2025). Solubility of mimetite Pb5 (AsO4) 3Cl–Vanadinite Pb5 (VO4) 3Cl solid solution series at 5–65° C. *Chemical Geology,**675,* 122609.

[CR86] Sprynskyy, M., Buszewski, B., Terzyk, A. P., & Namieśnik, J. (2006). Study of the selection mechanism of heavy metal (Pb^2+^, Cu^2+^, Ni^2+^, and Cd^2+^) adsorption on clinoptilolite. *Journal of Colloid and Interface Science,**304*(1), 21–28. 10.1016/j.jcis.2006.07.068 16989853

[CR87] Stefánsson, A. (2007). Iron (III) hydrolysis and solubility at 25 C. *Environmental Science & Technology,**41*(17), 6117–6123. 10.1021/es070174h 17937290

[CR88] Stępień, E., Manecki, M., & Bajda, T. (2025). Mimetite precipitation on Pb-modified zeolite: an effective approach for arsenate removal from water. *Mineralogia,**56*, 44–51.

[CR89] Stober, I., Grimmer, J. C., & Kraml, M. (2025). Origin and development of the geothermal fluids of the Baden-Baden area (SW-Germany): Implications for geothermal systems of granitic reservoirs. *Swiss Journal of Geosciences,**118*(1), 8.

[CR90] Stumm, W., & Morgan, J. J. (2013). *Aquatic chemistry: Chemical equilibria and rates in natural waters*. John Wiley & Sons.

[CR91] Syam Babu, D., & Nidheesh, P. V. (2021). A review on electrochemical treatment of arsenic from aqueous medium. *Chemical Engineering Communications,**208*(3), 389–410.

[CR92] Szlachta, M., & Wójtowicz, P. (2016). Treatment of arsenic-rich waters using granular iron hydroxides. *Desalination and Water Treatment,**57*(54), 26376–26381.

[CR93] Turek, P., Bajda, T., & Manecki, M. (2015). Dissolution of mimetite Pb5 (AsO4) 3Cl in malic acid solutions. *Mineralogia,**45*(1–2), 3.

[CR94] Twidwell, L. G., Robins, R. G., & Hohn, J. W. (2005). The removal of arsenic from aqueous solution by coprecipitation with iron (III). Arsenic metallurgy, 3–24.

[CR95] Velarde, L., Nabavi, M. S., Escalera, E., Antti, M. L., & Akhtar, F. (2023). Adsorption of heavy metals on natural zeolites: A review. *Chemosphere,**328*, Article 138508. 10.1016/j.chemosphere.2023.138508 36972873

[CR96] Vithanage, M., Dabrowska, B. B., Mukherjee, A. B., Sandhi, A., & Bhattacharya, P. (2012). Arsenic uptake by plants and possible phytoremediation applications: A brief overview. *Environmental Chemistry Letters,**10*(3), 217–224.

[CR97] Wang, A. O., Ptacek, C. J., Wilson, D., & Blowes, D. W. (2025). Geochemical stability of vitrified glass prepared from arsenic trioxide roaster waste from the Giant Gold Mine (Yellowknife, NT). *Journal of Hazardous Materials,**493*, Article 138098. 10.1016/j.jhazmat.2025.138098 40286669

[CR98] Wang, L., Putnis, C. V., Ruiz-Agudo, E., King, H. E., & Putnis, A. (2013). Coupled dissolution and precipitation at the cerussite-phosphate solution interface: Implications for immobilisation of lead in soils. *Environmental Science & Technology,**47*(23), 13502–13510. 10.1021/es4041946 24228938

[CR99] Wang, S., Gao, B., Li, Y., Creamer, A. E., & He, F. (2017). Adsorptive removal of arsenate from aqueous solutions by biochar supported zero-valent iron nanocomposite: Batch and continuous flow tests. *Journal of Hazardous Materials,**322*, 172–181. 10.1016/j.jhazmat.2016.01.052 26852252

[CR100] Wang, Y., Lv, C., Xiao, L., Fu, G., Liu, Y., Ye, S., & Chen, Y. (2019). Arsenic removal from alkaline leaching solution using Fe (III) precipitation. *Environmental Technology,**40*(13), 1714–1720. 10.1080/09593330.2018.1429495 29345188

[CR101] Staatsbad Wildbad (2026) Chemical composition of the Bad Wildbad thermal water. Available at: Staatsbad Wildbad GmbH mit Palais Thermal und Vital Therme Bad Wildbad (accessed 28 July 2026).

[CR105] Wudarska, A., Sordyl, J., Manecki, M., Zawila, A., & Bajda, T. (2022). Vibrational spectroscopic study of synthetic analogs of schultenite PbHAsO_4_- “phosphoschultenite” PbHPO_4_ solid solution series. *Polyhedron,**211*, 115534.

[CR106] Xie, J., Li, C., Chi, L., & Wu, D. (2013). Chitosan modified zeolite as a versatile adsorbent for the removal of different pollutants from water. *Fuel,**103*, 480–485.

[CR107] Xu, Y., & Schwartz, F. W. (1994). Lead immobilisation by hydroxyapatite in aqueous solutions. *Journal of Contaminant Hydrology,**15*(3), 187–206.

[CR108] Zhang, D., Wang, S., Wang, Y., Gomez, M. A., & Jia, Y. (2019). The long-term stability of calcium arsenates: Implications for phase transformation and arsenic mobilization. *Journal of Environmental Sciences,**84*, 29–41. 10.1016/j.jes.2019.04.017 31284914

[CR109] Zhu, Y. N., Zhang, X. H., Xie, Q. L., Wang, D. Q., & Cheng, G. W. (2006). Solubility and stability of calcium arsenates at 25° C. *Water, Air, and Soil Pollution,**169*(1), 221–238.

