# Supplementary material for: Arsenate immobilisation and mimetite formation on Pb-modified zeolite under variable aqueous chemistry
Source: Environ Geochem Health. 2026 Sep 14;48(15):591. doi: 10.1007/s10653-026-03472-6 (PMC13574900; doi:10.1007/s10653-026-03472-6)
Supplement: Supplementary file 1 [file 10653_2026_3472_MOESM1_ESM.docx]

**Fluid-mineral mediated formation of mimetite Pb₅(AsO₄)₃Cl for efficient arsenate removal from water using Pb-modified zeolite**

**Supplementary materials**

**Ewa Stępień^1,[1]^, Frederik Dunkel^2,[2]^, Maciej Manecki^1,[3]^, Tomasz Bajda^1,╃^**

1. **Faculty of Geology, Geophysics and Environmental Protection, AGH University of Krakow, al. Mickiewicza 30, 30-059 Kraków, Poland**
2. **Chair of Geology and Economic Geology, Department Applied Geosciences and Geophysics, Montanuniversität Leoben, Peter-Tunner-Straße 5, 8700 Leoben, Austria**

**Materials and methods**

**Modification of zeolite by Pb sorption**

Zeolite rock obtained from the Bystré deposit (Slovak Republic) and supplied by ZeoCem was used for experiments. X-ray powder diffraction (XRPD) analysis confirmed that the material consisted primarily of clinoptilolite, which constituted the dominant crystalline phase, accompanied by minor amounts of quartz, illite, and orthoclase as accessory minerals (Fig. SM 1). Previous studies have provided a thorough characterization of this material (e.g. Wołowiec et al., 2017; Solińska & Bajda, 2022).

To convert the zeolite from its natural calcium form into the sodium-exchanged form, it was first treated with a 2 M NaCl solution for 24 hours. The details of Pb-modified zeolite preparation by sorption are described separately (Stępień et al., 2025). Briefly, the sodium-exchanged zeolite was converted into Pb-modified zeolite by reacting it with a solution containing 4000 mg Pb/l. After sorption, the material was thoroughly rinsed with redistilled water to eliminate residual Pb²⁺ and desorb weakly bound ionic species. Washing and centrifugation (at 4,500 rpm for 5 minutes) was repeated seven times until Pb concentration in the supernatant fell below the detection limit (0.01 mg Pb/L). Air-dried powder was used for the further experiments. Lead content in a modified zeolite, as determined by acid digestion (microwave mineralization in a mixture of HF and HCl with few drops of HClO_4_), was equal to 70 g Pb per kg of dry zeolite. The Pb loading represents the composition of the intentionally synthesized reactive material and does not correspond to Pb concentrations encountered in natural environmental media.

Pb modification resulted in only minor changes in the surface and porosity properties determined by BET analysis (Tab. SM 1). A slight decrease in the specific surface area was observed after Pb modification, while the remaining pore parameters remained nearly unchanged. These results indicate that Pb incorporation had a limited effect on the textural properties of the zeolite and did not significantly alter its pore structure.

Textural characteristics were evaluated based on low-temperature N_2_ adsorption-desorption isotherms recorded at -196 °C using a NOVA 800 sorption analyser (Anton Paar) within a relative pressure range of approximately 10^-3^ to 0.99. Prior to analysis, the samples were degassed under vacuum at 105 °C for 12 h. The specific surface area was determined using the BET (Brunauer-Emmett-Teller) method, whereas the total pore volume was derived at p/p_0_ ≈ 0.99. Textural parameters were determined according to ISO regulations for pore size distribution analysis. The applied methodology was also consistent with NIST recommendations for porosity and specific surface area measurements. Mesopore volume and pore size distribution were determined using the NLDFT method with the N₂@77 K silica/zeolite cylindrical pore adsorption model implemented in Kaomi 2.0 software (Anton Paar).

**Supplementary figures**

Figure SM 1. XRD patterns of natural Na-transformed zeolite (clinoptilolite) enriched in admixtures of orthoclase (O), illite (I), and quartz (Q), and zeolite after modification through sorption of Pb(II) (Pb-clinoptilolite).

**A.**

**B.**

Figure SM 2. **(A)** Map of the water sampling location in Germany (Bad Wildbad, Baden-Württemberg) and (**B)** the exterior photograph of the Vital Therme technical facility.

**A.**

**B.**

Figure SM 3. **(A)** Map of the water sampling location in Poland (Złoty Stok, in the vicinity of Kłodzko; Lower Silesia region) and **(B)** the photograph of the sampling location.

**A.**

**B.**

**C.**

Figure SM 4. **A.** Map of the water sampling location in Austria (Upper Mur Valley, in the vicinity of Knittelfeld; Styria region) and the photograph of the sampling location (**B** – Upper Mur Valley A; **C** – Upper Mur Valley B).

Figure SM 5. SEM (I.), EDS (II.), and XRPD (III.) results for Pb-modified zeolite reacted with arsenate solutions (0.1-50 mg As(V)/L, respectively I. A-D and II. A-D) at initial pH 2, in the presence of 20 mg/L Cl^-^. Compared to pH 6, mimetite precipitation was strongly reduced at low initial arsenate concentrations, with only sparse, fine-grained precipitates visible on SEM images and weak or absent reflections in XRPD patterns (I. A-C and III. 0.1 – 5 mg As(V)/L). At the highest concentration (I. D, 50 mg As(V)/L), however, precipitation proceeded similarly to pH 6 (Fig. 2 D), indicating that sufficient arsenate supply could overcome the reduced stability of mimetite under acidic conditions. EDS spectra were collected from the areas shown in panel I. A–D, and acquired by wide-area scans covering the entire SEM fields of view.

Figure SM 6. Characterization of Pb-zeolite after As(V) removal in the presence of accompanying cations. (I) XRPD patterns showing the formation of mimetite Pb5(AsO4)3Cl after Pb-zeolite exposure to arsenate and chloride solution (50 mg As(V)/L and 20 mg Cl^-^/L) for 7 days, with variations depending on the presence of different cations (Cd, Zn, Cu, Ni, Mn, Ca, K, Al, Mg; molar ratio 1:1). (II) SEM–EDS spectra showing elemental composition of precipitates formed under the same conditions confirming formation of lead chloro-arsenate. (III) SEM micrographs illustrating surface morphology of Pb-zeolite after As(V) treatment in the presence of different cations, highlighting formation of mimetite incrustations. EDS spectra were collected from the areas shown in panel III, and acquired by wide-area scans covering the entire SEM fields of view.

Figure SM 7. Results of synthesis experiments from mixed Pb-As-PO₄-Cl solutions at pH 6. (A) SEM image showing the fine precipitate matrix; (B) SEM image highlighting a larger crystalline aggregate; (C) X-ray diffraction pattern of the recovered solid with reference reflections for mimetite (Pb_5_(AsO_4_)_3_Cl) and pyromorphite (Pb_5_(PO_4_)_3_Cl); (D) EDS spectrum of the crystalline phase, confirming the presence of Pb, As, P, Cl, and O. Spectrum was obtained by a smaller-area scan of the region marked by the red rectangle. The results indicated the formation of mimetite-pyromorphite type phases, suggesting either a solid solution or a mixture of the two end-members.

**Supplementary tables**

Table SM 1. Textural characteristics of natural and Pb-modified zeolite determined by nitrogen adsorption-desorption, showing BET surface area, total pore volume, micropore volume, and mesopore volume.

| **Property** | **Natural zeolite** | **Pb-modified zeolite** |
| --- | --- | --- |
| BET surface area [m^2^/g] | 28.819 | 24.838 |
| BET total pore volume [cm^3^/g] | 0.0993 | 0.1001 |
| BET micropores volume [cm^3^/g] | 0.0015 | 0.0006 |
| BET mesopores volume [cm^3^/g] | 0.0874 | 0.0860 |

Table SM 2. PHREEQC input script and simulation results for the dissolution of mimetite (Pb_5_(AsO_4_)_3_Cl) in NaCl solution (20 mg Cl^-^/L; pH 2 and 6) and redistilled water (pH 6). Geochemical modelling was performed using PHREEQC (version 3.8.6.17100; Parkhurst & Appelo, USGS) with the modified llnl2023.dat thermodynamic database. The solubility product (Ksp) of mimetite was included based on values reported by Bajda (2010).

```
# 1.
SOLUTION 1 NaCl_solution
temp 25
units mg/L
pH 6.0
pe 4.0
Na 12.97    # 20 mg Cl/L z NaCl
Cl 20
# No As and Pb in the solution
# ------------------------------------------
# Equilibrium of the solid phase
EQUILIBRIUM_PHASES 1
MimetiteCl 0.0 1000.0
CO2(g) -3.5
# Fix_H+ -6.0 HNO3
# ------------------------------------------
# Output
SELECTED_OUTPUT
-file selected_output_dissolution in NaCl.txt
-totals Pb Cl As

END

# 2.
PHASES
Mimetite
Pb5(AsO4)3Cl = 5 Pb+2 + 3 AsO4-3 + Cl-
# Sodium chloride solution
# ------------------------------------------
SOLUTION 1 NaCl_solution
temp 25
units mg/L
pH 2.0
pe 4.0
Na 12.97    # 20 mg Cl/L z NaCl
Cl 20
# No Pb and As in the solution
# Equilibrium of the solid phase
# ------------------------------------------
EQUILIBRIUM_PHASES 1
MimetiteCl 0.0 1000.0
CO2(g) -3.5
# Fix_H+ -2.0 HNO3
# Output
# ------------------------------------------
SELECTED_OUTPUT
-file selected_output_dissolution in NaCl pH2.txt
-totals Pb Cl As

END
```

```
# 3.
# Equilibrium dissolution of mimetite in redistilled water

PHASES
Mimetite
    Pb5(AsO4)3Cl = 5 Pb+2 + 3 AsO4-3 + Cl-

# ------------------------------------------
# Redistilled water
# ------------------------------------------
SOLUTION 1 redistilled water
    temp 25
    units mg/L
    pH 6.0
    pe 4.0
# Equilibrium of the solid phase
# ------------------------------------------
EQUILIBRIUM_PHASES 1
    MimetiteCl 0.0 1000.0   # dissolution/precipitation of mimetite
    CO2(g) -3.5           # equilibrium with air
# Output
# ------------------------------------------
SELECTED_OUTPUT
    -file selected_output_dissolution in redistilled water.txt
    -totals Pb Cl As

END
```

Table SM 3. Modelled equilibrium concentrations and speciation of Pb and As for mimetite (Pb₅(AsO₄)₃Cl) dissolution at pH 6 in redistilled water, and at pH 2 and pH 6 in NaCl solution (20 mg Cl^-^/L). Concentrations are reported in mg/L, and arsenate speciation shows the fraction of total As(V) in each form. Values are presented as single measurements; error estimates were not calculated for these data.

| **pH** | **Solution** | **Pb [mg/L]** | **As(V) [mg/L]** | **Dominant As species** | **% H₃AsO₄** | **% H₂AsO₄⁻** | **% HAsO₄²⁻** | **% AsO₄³⁻** |
| --- | --- | --- | --- | --- | --- | --- | --- | --- |
| **6** | **Redistilled water** | 0.216 | 0.047 | H₂AsO₄⁻/ HAsO₄²⁻ | ~0% | 93% | 7% | ~0% |
| **6** | **NaCl 20 mg Cl^-^/L** | 0.098 | 0.021 | H₂AsO₄⁻/HAsO₄²⁻ | ~0% | 93% | 7% | ~0% |
| **2** | **NaCl 20 mg Cl^-^/L** | 77.700 | 16.965 | H₃AsO₄/H₂AsO₄⁻ | 60% | 34% | ~0% | ~0% |

Table SM 4. Initial measured concentrations of accompanying cations (mg/L) at different molar ratios to arsenate solutions with initial [As(V)] = 5 mg/L and 50 mg/L.

| **Accompanying cation** | **Initial [As(V)]**  **5 mg/L** | | | **Initial [As(V)]**  **50 mg/L** |
| --- | --- | --- | --- | --- |
|  | **As/cation molar ratio** | | | |
|  | **1:1** | **1:2** | **2:1** | **1:1** |
| **Mg** | 1.50 | 3.00 | 0.52 | 15.00 |
| **Al** | 1.80 | 2.88 | 0.76 | 18.00 |
| **K** | 2.50 | 5.20 | 1.30 | 25.00 |
| **Ca** | 2.50 | 5.00 | 1.03 | 25.00 |
| **Mn** | 2.95 | 7.10 | 1.62 | 29.50 |
| **Ni** | 3.31 | 7.71 | 2.25 | 33.10 |
| **Cu** | 4.16 | 8.45 | 2.05 | 41.60 |
| **Zn** | 4.94 | 7.94 | 2.99 | 49.40 |
| **Cd** | 7.02 | 14.10 | 4.57 | 70.20 |

Table SM 5. Initial measured concentrations of accompanying anions (mg/L) at different molar ratios to arsenate solutions with initial [As(V)] = 5 mg/L and 50 mg/L.

| **Initial [As(V)] 5 mg/L** | | | |
| --- | --- | --- | --- |
| **Accompanying anion** | **As/anion molar ratio** | | |
|  | **1:1** | **1:2** | **2:1** |
| **F^-^** | 1.20 | 2.54 | 0.65 |
| **SO_4_^2-^** | 6.50 | 12.00 | 3.00 |
| **PO_4_^3-^** | 6.30 | 12.00 | 3.10 |
| **Initial [As(V)] 50 mg/L** | | | |
| **Accompanying anion** | **As/anion molar ratio** | | |
|  | **1:1** | | |
| **F^-^** | 12.00 | | |
| **SO_4_^2-^** | 65.00 | | |
| **PO_4_^3-^** | 63.00 | | |

Table SM 6. Chemical composition of natural waters used in the experiments simulating the natural environment (n.a. – not analysed).

The chemical analysis of the Bad Wildbad water (Vital Therme, Germany) was provided by the technical facility at the sampling site. Water samples from Złoty Stok (Trująca River, Poland) and the Upper Mur Valley (Austria) were analysed by the authors using ICP-MS/OES techniques and UV-Vis spectrophotometry. HCO₃⁻ was calculated from alkalinity.

| **Water sample** | **Bad Wildbad (Vital Therme, Germany)** | **Złoty Stok (Trująca River, Poland)** | **Upper Mur Valley A (Austria)** | **Upper Mur Valley B (Austria)** |
| --- | --- | --- | --- | --- |
| pH | 7.6 | 7.3 | 7.0 | 7.4 |
| RDO [mg/L] | n.a. | n.a. | 8.47 | 1.33 |
| **Ion** | **Concentration [mg/L]** | | | |
| Na⁺ | 136.00 | 4.87 | 4.85 | 6.25 |
| K⁺ | 7.31 | 1.38 | 5.28 | 6.37 |
| NH₄⁺ | <0.04 | n.a. | n.a. | n.a. |
| Mg²⁺ | 3.12 | 11.07 | 12.12 | 22.88 |
| Ca²⁺ | 37.00 | 38.26 | 47.15 | 34.96 |
| Mn²⁺ | <0.003 | <0.005 | <0.005 | <0.005 |
| Fe²⁺/³⁺ | <0.01 | <0.01 | 0.13 | 0.08 |
| Ba²⁺ | 0.08 | <0.01 | 0.04 | 0.02 |
| Cu²⁺ | 0.02 | <0.005 | 0.01 | <0.001 |
| As⁵⁺ | 0.14 | 0.77 | 0.24 | 0.38 |
| F⁻ | 1.05 | n.a. | 0.27 | 0.35 |
| Cl⁻ | 140.60 | 5.70 | 1.44 | 3.27 |
| NO₃⁻ | 1.54 | n.a. | 0.024 | 0.023 |
| SO₄²⁻ | 30.30 | 29.20 | 57.00 | 33.00 |
| HCO₃⁻ | 227.20 | 149.10 | 205.00 | 221.00 |
| PO₄³⁻ | 0.04 | 0.05 | <0.01 | <0.01 |
| BO_3_³⁻ | n.a. | 0.54 | n.a. | n.a. |

Table SM 7. Lead concentrations (mg/L) released from Pb-zeolite after 24 h and 7 days at pH 6 in NaCl solutions: (A) Na⁺ = 13 mg/L, Cl⁻ = 20 mg/L; (B) Na⁺ = 16.1 mg/L, Cl⁻ = 25 mg/L, including ~3.1 mg/L Na⁺ equivalent to 5 mg As(V)/L as Na₂HAsO₄·7H₂O. Values are presented as single measurements; error estimates were not calculated for these data.

| **Time** | **Pb concentration [mg/L]** | |
| --- | --- | --- |
|  | **A.** | **B.** |
| **24 hours** | 0.291 | 0.194 |
| **7 days** | 0.065 | 0.122 |

1. Corresponding author. E-mail address: estepien@agh.edu.pl (Ewa Stępień)
2. E-mail address: frederik.dunkel@unileoben.ac.at (Frederik Dunkel)
3. E-mail address: gpmmanec@cyf-kr.edu.pl (Maciej Manecki)

   ^╃^ E-mail address: bajda@agh.edu.pl (Tomasz Bajda)
